# TAD-like single-cell domain structures exist on both active and inactive X chromosomes and persist under epigenetic perturbations

**DOI:** 10.1186/s13059-021-02523-8

**Published:** 2021-11-08

**Authors:** Yubao Cheng, Miao Liu, Mengwei Hu, Siyuan Wang

**Affiliations:** 1 Department of Genetics, Yale School of Medicine, Yale University, New Haven, CT 06510 USA; 2 Department of Cell Biology, Yale School of Medicine, Yale University, New Haven, CT 06510 USA; 3 Yale Combined Program in the Biological and Biomedical Sciences, Yale University, New Haven, CT 06510 USA; 4 Molecular Cell Biology, Genetics and Development Program, Yale University, New Haven, CT 06510 USA; 5 Biochemistry, Quantitative Biology, Biophysics and Structural Biology Program, Yale University, New Haven, CT 06510 USA; 6 M.D.-Ph.D. Program, Yale University, New Haven, CT 06510 USA; 7 Yale Center for RNA Science and Medicine, Yale University School of Medicine, New Haven, CT 06510 USA; 8 Yale Liver Center, Yale University School of Medicine, New Haven, CT 06510 USA

**Keywords:** Topologically associating domain (TAD), TAD-like structure, Single-cell domain, X chromosome, X inactivation, Chromatin folding, Chromatin compaction, Image-based spatial genomics, 3D genomics, Chromatin tracing, Multiplexed sequential fluorescence in situ hybridization (FISH)

## Abstract

**Background:**

Topologically associating domains (TADs) are important building blocks of three-dimensional genome architectures. The formation of TADs has been shown to depend on cohesin in a loop-extrusion mechanism. Recently, advances in an image-based spatial genomics technique known as chromatin tracing lead to the discovery of cohesin-independent TAD-like structures, also known as single-cell domains, which are highly variant self-interacting chromatin domains with boundaries that occasionally overlap with TAD boundaries but tend to differ among single cells and among single chromosome copies. Recent computational modeling studies suggest that epigenetic interactions may underlie the formation of the single-cell domains.

**Results:**

Here we use chromatin tracing to visualize in female human cells the fine-scale chromatin folding of inactive and active X chromosomes, which are known to have distinct global epigenetic landscapes and distinct population-averaged TAD profiles, with inactive X chromosomes largely devoid of TADs and cohesin. We show that both inactive and active X chromosomes possess highly variant single-cell domains across the same genomic region despite the fact that only active X chromosomes show clear TAD structures at the population level. These X chromosome single-cell domains exist in distinct cell lines. Perturbations of major epigenetic components and transcription mostly do not affect the frequency or strength of the single-cell domains. Increased chromatin compaction of inactive X chromosomes occurs at a length scale above that of the single-cell domains.

**Conclusions:**

In sum, this study suggests that single-cell domains are genome architecture building blocks independent of the tested major epigenetic components.

**Supplementary Information:**

The online version contains supplementary material available at 10.1186/s13059-021-02523-8.

## Background

Genomic DNA is compactly folded into the cell nucleus and is spatially organized with other nuclear components in eukaryotic cells [1–8]. This folding and spatial organization has been shown to control many important genomic functions, and altered organization has been linked to diseases [1–11]. Recent studies have identified topologically associating domains (TADs, also known as contact domains) as an important organizational unit of chromatin folding [12–16]. TADs are continuous sections of the genome at a length scale of around tens to hundreds of kilobases (kb) with enhanced self-interactions, initially discovered by high-throughput versions of population-averaged chromosome conformation capture methods (e.g., Hi-C [3, 17]). Imaging studies showed that, in real space, individual TADs are physically separated, though adjacent TADs may partially overlap [13, 18–21]. TADs are implicated in defining the scope of promoter-enhancer interactions [22, 23]. Mechanistically, at least a subset of TADs are established through a loop-extrusion mechanism, in which cohesin dynamically extrudes a chromatin loop until the cohesin motor is blocked by CCCTC-binding factor (CTCF) bound to the DNA in an effective orientation [24–27]. Here the chromatin loop forms a TAD and the CTCF binding site marks the TAD boundary. In addition, TAD boundaries at least occasionally coincide with the boundaries of epigenetic domains along the genome [12, 13].

At the single-cell or single-chromosome-copy level, TADs are highly variant structures. Single-cell sequencing studies showed that the TADs identified from population averaged Hi-C maps do not form in every cell [28, 29]. Recently, an image-based spatial genomics study using chromatin tracing [30, 31], a highly multiplexed sequential DNA fluorescence in situ hybridization (FISH) technique, showed that TAD boundaries on human chromosome 21 are highly variant from one copy of the chromosome to another [32]. To distinguish from the population averaged TAD structures, these highly variant single-cell structures were named as TAD-like structures [32], later also named as single-cell domains [33]. Strikingly, single-cell domains persisted in single chromosomes even after the removal of key loop-extrusion machinery and after the disappearance of TAD structures at the population level [32]. Recent computational modeling studies suggested that dynamic chromatin contacts captured by epigenetic interactions may establish the single-cell domains [34, 35]. Following this hypothesis, we reason that the active and inactive X chromosomes of a female cell may show distinct features of single-cell domains, given the drastically different epigenetic states of the two chromosomes despite largely identical sequences [36–38]. Allele-specific Hi-C studies showed that the inactive X chromosome is largely devoid of TAD structures, while the active X chromosome contains TADs [39, 40]. It is unclear whether single-cell domains exist on both X chromosomes, and whether the single-cell domains are affected by changes in the epigenetic states. To answer these questions, here we perform chromatin tracing on the active and inactive X chromosomes in human cell lines. Our results show that single-cell domains are present on both active and inactive X chromosomes at comparable frequencies and strengths. We made this same observation in cells of distinct biological backgrounds. Chemical perturbations of several major epigenetic components and transcription do not appear to greatly affect the single-cell domains. In addition, by comparing the fine-scale chromatin tracing data in this work with our previous work reporting larger scale chromatin traces, we also show that the increased compaction of the inactive X chromosome in comparison to the active X chromosome occurs at a length scale above that of single-cell domains. In sum, these observations offer a single-cell view of the principles of X chromosome folding and compaction and suggest that the mechanism underlying the establishment and/or maintenance of single-cell domains may not involve the tested major epigenetic components.

## Results

**Fig. 1:**
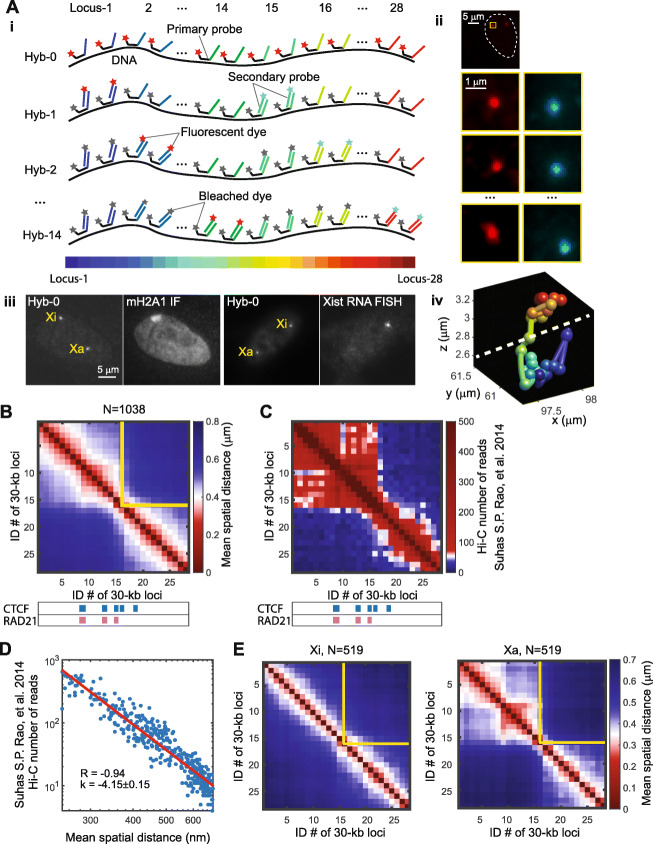
Chromatin tracing reveals the fine folding architecture of inactive and active X chromosomes. (Target region: ChrX: 76,800,000–77,640,000, hg18). **A** Schematic illustration of the chromatin tracing approach (i), raw images of the chromatin tracing (ii), co-immunofluorescence of mH2A1 and co-RNA FISH of *XIST* (iii), and a reconstructed chromatin trace showing two domains separated by a white dashed line (iv). In ii, the white dashed line in the top panel outlines a cell nucleus. The yellow boxed region containing a copy of the targeted chromatin region is shown in the lower panels of ii. The four rows of panels correspond to Hybs-0, 1, 2, and 14 as indicated on the left. **B** Mean spatial distance matrix of the traced genomic region calculated from pooled X chromosome copies including both active and inactive X’s in IMR-90 cells. **C** Hi-C contact frequency matrix of the same genomic region as in **B**. CTCF and RAD21-binding peaks are illustrated in blue and pink respectively. **D** Comparison of mean spatial distance from chromatin tracing with contact frequency measured by Hi-C. **E** Separate mean spatial distance matrices from inactive (left) and active (right) X chromosomes. The yellow lines in **B** and **E** represent the ensemble TAD boundary

To investigate the chromatin folding of active and inactive X chromosomes (Xa and Xi) at the TAD-length scale in single cells, we used chromatin tracing [30, 31] to trace the chromatin folding organization of an 840-kb region of the X chromosome in a human female IMR-90 lung fibroblast cell line. This selected 840-kb region spans a TAD boundary, based on previously published IMR-90 Hi-C datasets [12, 16]. We partitioned this region of interest into consecutive 30-kb segments, labeled each segment with 400 primary oligonucleotide probes, and imaged each segment following our previously established chromatin tracing protocol [30, 41]. In brief, we first labeled and imaged the entire region with a library of dye-labeled primary oligonucleotide probes. Each primary probe contains a targeting sequence that hybridizes to the genomic DNA, and an overhanging readout sequence that is unique to each 30-kb segment (Fig. 1A). We then sequentially hybridized to the sample dye-labeled secondary probes with sequences complementary to the readout sequences on the primary probes, allowing each 30-kb segment to be sequentially visualized using three-dimensional (3D) epifluorescence microscopy (Fig. 1A). We performed two-color imaging to simultaneously visualize two segments at a time and then photobleached the sample before the next round of sequential hybridization. This hybridization-imaging-bleaching procedure was repeated until all secondary probes were applied and all chromatin segments were imaged (Fig. 1A). The center positions of all segments were individually measured and linked based on their order on the genomic map to reconstruct the 3D folding conformation of the region in single copies of X chromosomes in single cells (Fig. 1A). To distinguish Xa and Xi, we performed either co-immunofluorescence targeting macroH2A.1 (mH2A1), a histone variant enriched on Xi, or co-RNA FISH targeting *XIST* long noncoding RNA (Fig. 1A).

To validate the chromatin traces, we calculated a mean spatial distance matrix with matrix elements representing the mean spatial distances between pairs of the targeted 30-kb segments and compared these distances from imaging with the corresponding contact frequencies from published ensemble IMR-90 Hi-C data [16]. The mean spatial distance matrix displayed a TAD boundary at the same genomic position as in the ensemble Hi-C contact frequency matrix (Fig. 1B, C). The domain boundary location on the genome was enriched with CTCF and cohesin (represented by one of the core subunits RAD21) binding (Fig. 1B, C), as profiled by chromatin immunoprecipitation sequencing (ChIP-seq) [42]. The mean spatial distance measurements were highly correlated with the Hi-C contact frequencies, with a correlation coefficient of − 0.94 (Fig. 1D). A power-law fitting showed that the Hi-C contact frequency was inversely proportional to the 4^th^ power of the mean spatial distance (Fig. 1D), similar to the power-law relationship previously observed for autosome chromatin folding [30, 32, 33, 43]. These observations indicated that our chromatin tracing results were consistent with Hi-C results at the ensemble level, and provided a validation of the chromatin tracing data.

Next, to compare the chromatin folding conformations between Xi and Xa, we generated separate mean spatial distance matrices for Xi and Xa. The Xi matrix showed an attenuation of the major TAD boundary, but some residual TAD features still persisted, while the Xa matrix showed a prominent TAD boundary at the same coordinate as the whole-population TAD boundary (Fig. 1E). This observation is consistent with previous allele-specific Hi-C studies showing that TADs are largely attenuated on Xi [39, 40, 44], likely due to a global loss of cohesin along Xi [39]. This consistency supports the accuracy of our identification of Xi versus Xa.

**Fig. 2:**
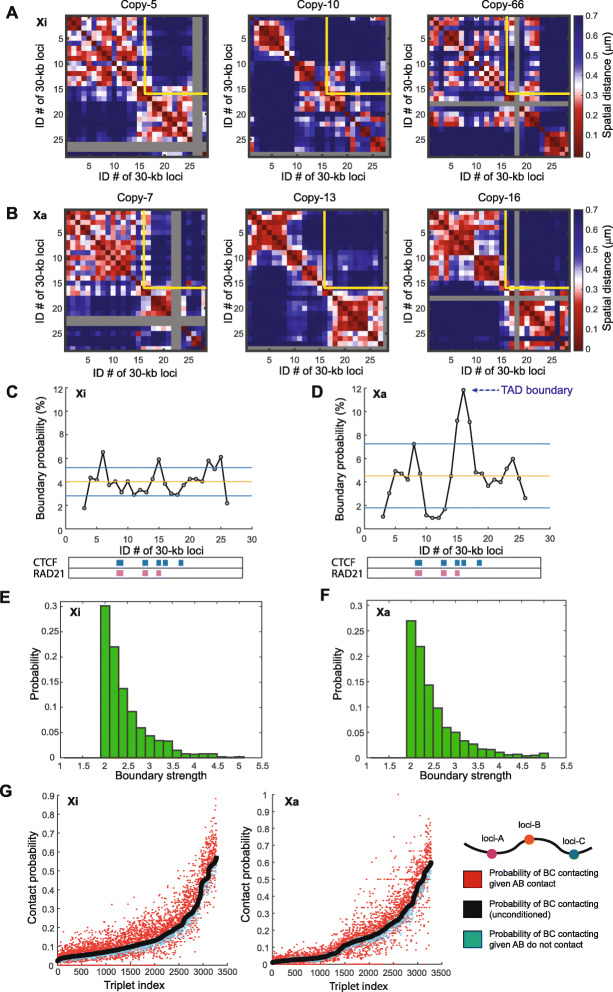
Highly variant TAD-like single-cell domains are present on both inactive and active X chromosomes in IMR-90 cells (target region as stated in Fig. 1). **A** Examples of individual spatial distance matrices from single copies of inactive X chromosomes. **B** Examples of individual spatial distance matrices from single copies of active X chromosomes. Gray rows and columns in **A** and **B** indicate undetected loci. The yellow lines in **A** and **B** represent the ensemble TAD boundary. **C** The boundary probabilities of single-cell domains in inactive X chromosomes. **D** The boundary probabilities of single-cell domains in active X chromosomes. Note the higher boundary probability at the population averaged TAD boundary (arrow). The horizontal lines in **C** and **D** represent the mean probabilities (orange) and plus/minus one standard deviation (blue). CTCF and RAD21-binding peaks are illustrated in blue and red respectively. **E** The boundary strengths of single-cell domains in inactive X chromosomes. **F** The boundary strengths of single-cell domains in active X chromosomes. **G** Cooperativity of chromatin interaction in inactive and active X chromosomes. For all ordered triplets in the traced chromatin region, contact probabilities (defined with 200-nm proximity threshold) of genomic loci B and C given contact/non-contact of genomic loci A and B are shown in red and blue, respectively. “Ordered” means that the locus numbers of A, B, and C are in an ascending order. The unconditional contact probabilities between loci B and C are shown in black. The triplet indices are arranged in an ascending order based on the unconditional contact probabilities between loci B and C. *N* = 483 for **C**, **E**, and the left panel of **G**. *N* = 477 for **D**, **F**, and the right panel of **G**

To detect potential single-cell domains in individual copies of X chromosomes, we calculated the individual spatial distance matrices for single copies of Xi and Xa and found that individual Xi and Xa copies both displayed single-cell domains with sharp boundaries (Fig. 2A, B). The boundary positions of the single-cell domains were highly variant among individual copies of Xi and Xa (Fig. 2A–D). Quantification of boundary probabilities along the traced genomic region showed that in Xi, the single-cell domain boundaries had significantly more uniform distribution across the traced region (*p* = 2.09 × 10^−4^, *F*-test) (Fig. 2C, D), leading to the lack of strong ensemble boundary in Xi (Fig. 1E), as the single-cell domain boundaries across this genomic region are “averaged out” during the ensemble calculation. In Xa, however, single-cell domain boundaries preferentially resided at the ensemble TAD boundary (Fig. 2D), leading to a prominent ensemble boundary after population averaging (Fig. 1E). Quantifications of the boundary strengths showed that the boundaries of single-cell domains in Xi and Xa are similarly strong (Fig. 2E, F). The single-cell domains reflect prevalent cooperativity of chromatin interaction [32]. To measure this cooperativity, we compared the conditional and unconditional contact probabilities among triplets of chromatin segments, using 200 nm as the threshold of contact: Given each combination of ordered chromatin segments A, B and C, we measured the unconditional contact probability between B and C and compared that with the conditional contact probability between B and C given that A and B were making contact, as well as the conditional contact probability between B and C given that A and B were not making contact. The data showed that in both Xi and Xa, the conditional probability of B-C contact given A-B contact is systematically higher than the unconditional probability, and higher than the conditional probability without A-B contact (Fig. 2G). These results indicate that both Xi and Xa can contain single-cell domains of similar features.

To further analyze the structural differences between Xi and Xa, we performed Louvain-Jaccard clustering of individual chromatin traces, using each chromatin trace as a high-dimensional data entry and the pairwise distances between the 30-kb target regions as variables. We found that chromatin traces formed clusters with different conformations (Additional file 1: Fig. S1A-B). Each conformational cluster had a mixture of Xi and Xa traces, indicating that Xi and Xa both had similarly diverse conformations (Additional file 1: Fig. S1C-D). Clusters had different ratios of Xi and Xa traces (Additional file 1: Fig. S1D), consistent with the different ensemble chromatin conformations of Xi and Xa (Fig. 1E).

To study whether our findings can be generalized to other regions on the human X chromosome, we traced the chromatin folding of two additional 840-kb regions spanning TAD boundaries identified from Hi-C [12, 16]. One additional target region corresponded to a closed chromatin region with low signals in assay for transposase-accessible chromatin using sequencing (ATAC-seq) and high H3K9me3 ChIP-seq signals, whereas the other target region was an open chromatin region with high ATAC-seq signals and active gene transcription (Additional file 1: Fig. S2). To compare chromatin conformations of Xi and Xa at the ensemble level, we generated separate mean spatial distance matrices for Xi and Xa. Similar to the target region profiled above (Fig. 1E), the Xi matrices showed attenuated TAD boundaries while the Xa matrices showed prominent TAD boundaries in both the closed and open regions (Additional file 1: Fig. S3A, S4A). To compare potential single-cell domains in individual copies of Xi and Xa, we generated individual spatial distance matrices of single copies of Xi and Xa. Both individual copies of Xi and Xa traces showed single-cell domains with sharp boundaries in both the closed and open regions (Additional file 1: Fig. S3B-C, S4B-C). Single-cell domain boundary positions were highly variant in both Xi and Xa traces (Additional file 1: Fig. S3B-C, S4B-C). Quantifications of boundary probabilities suggested that single-cell domain boundaries in Xi were significantly more uniformly distributed along the traced regions than those in Xa (*p* = 0.026 for the closed region and *p* = 0.0098 for the open region, *F*-tests), whereas single-cell domain boundaries in Xa preferentially resided at the ensemble TAD boundaries (Additional file 1: Fig. S3D-E, S4D-E). Moreover, quantifications of boundary strengths indicated that Xi and Xa single-cell domains had similar boundary strengths (Additional file 1: Fig. S3F-G, S4F-G). We further performed Louvain-Jaccard clustering of individual chromatin traces from the two regions (Additional file 1: Fig. S5A, S6A) and identified chromatin trace clusters with different conformations (Additional file 1: Fig. S5B, S6B). Each conformational cluster contained a mixture of Xi and Xa traces (Additional file 1: Fig. S5C, S6C), indicating that Xi and Xa both had diverse conformations in the two additional regions. These analyses revealed that the organizational features of ensemble Xi and Xa chromatin and the existence of diverse single-cell domains are not limited to one X chromosome region.

To check the prevalence of the X chromosome single-cell domains in different cell backgrounds, we imaged another human female cell line, hTERT-RPE-1, derived from retina pigmented epithelium, and found that the above observations in IMR-90 lung fibroblast cells were conserved in the hTERT-RPE-1 cells: Xi showed attenuated TAD boundary whereas Xa showed strong ensemble TAD boundary (Additional file 1: Fig. S7A-B). Individual Xi and Xa traces showed similarly frequent and strong single-cell domains (Additional file 1: Fig. S7C-H). These observations show that the single-cell domains on Xi and Xa are not unique to the IMR-90 cells.

**Fig. 3:**
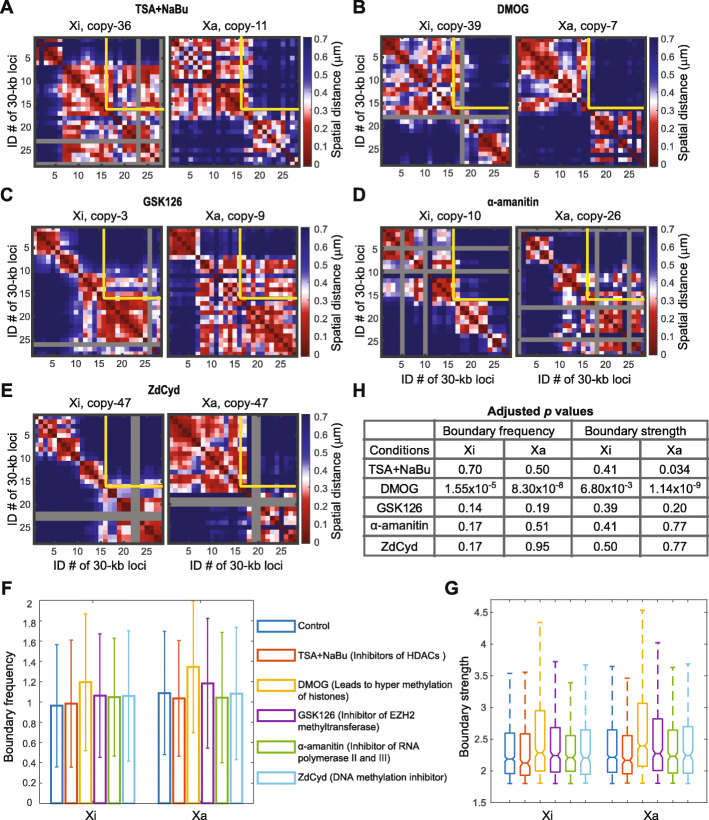
Single-cell domains persist under perturbations of epigenetic components and transcription machinery (target region as stated in Fig. 1). **A** Single-cell domains with HDAC inhibitor TSA + NaBu treatment (leading to hyper acetylation of histones). **B** Single-cell domains with DMOG treatment (leading to hyper methylation of histones). **C** Single-cell domains with GSK126 treatment (leading to lower H3K27me3). **D** Single-cell domains with α-amanitin treatment (leading to inhibition of transcription). **E** Single-cell domains with ZdCyd treatment (leading to inhibition of methylation of cytosine-C5 of DNA). Gray rows and columns in **A**–**E** indicate undetected loci. The yellow lines in **A**–**E** represent the ensemble TAD boundary. **F** Average boundary frequencies with or without TSA + NaBu, DMOG, GSK126, α-amanitin, and ZdCyd treatments. Error bars stand for standard deviation. **G** Boundary strengths with or without TSA + NaBu, DMOG, GSK126, α-amanitin, and ZdCyd treatments. For each box, the horizontal lines from top to bottom represent the non-outlier maximum, 75% quantile, median, 25% quantile, and non-outlier minimum. Outliers are defined as values that are more than 1.5 times the interquartile range away from the bottom or top of the box. In F and G from left to right: *N* = 483, 477, 182, 201, 248, 319, 186, 183, 163, 188, 111, 115. **H** A summary of all adjusted *p* values calculated in **F** and **G**. All *p* values were calculated using two-sided Student’s *t* tests with false discovery rate correction

Next, to test the hypothesis that epigenetic interactions underlie the formation of single-cell domains, we applied several epigenetic or transcription perturbations using commercially available small molecule inhibitors and measured their effects on the single-cell domains. First we treated IMR-90 cells for 24 h with a combination of histone deacetylase (HDAC) inhibitors trichostatin A (TSA) and sodium butyrate (NaBu) that lead to hyperacetylation of histones (Additional file 1: Fig.S8A), given that histone acetylation prevents nucleosome compaction [20, 45, 46]. Surprisingly, the single-cell domains persisted in both Xi and Xa under the drug treatment with similar boundary frequency and strength as in the untreated control (Fig. 3). We further treated IMR-90 cells for 12 h with dimethyloxalylglycine (DMOG)—an HIF-prolylhydroxylase inhibitor (Additional file 1: Fig. S8B), which causes a global increase of histone methylation [47]; for 48 h with GSK126, an enhancer of zeste homolog 2 (EZH2) methyltransferase inhibitor (Additional file 1: Fig. S8C); for 8 h with α-amanitin, an RNA polymerase inhibitor (Additional file 1: Fig. S8D); and for 24 h with 5-aza-2’-deoxycytidine (ZdCyd), a DNA methylation (cytosine-C5) inhibitor [48] (Additional file 1: Fig. S8E). Time course immunofluorescence measurements, intron RNA FISH, and co-immunofluorescence with DNA FISH confirmed the effectiveness of the perturbations in IMR-90 cells (Additional file 1: Fig. S8-9). The DMOG treatment led to a minimal increase of both the boundary frequency and boundary strength (Fig. 3F, G), whereas none of the other treatments significantly affected the appearance, boundary frequencies, or strengths of the single-cell domains in Xi or Xa (*p* > 0.01 with false discovery rate correction) (Fig. 3). The small increase of boundary frequency and strength under the DMOG treatment could be due to secondary effects of the drug. These observations suggest that several major epigenetic components, including histone acetylation and methylation, DNA methylation, and RNA polymerase, may not be the major factors determining the formation or maintenance of single-cell domains.

**Fig. 4:**
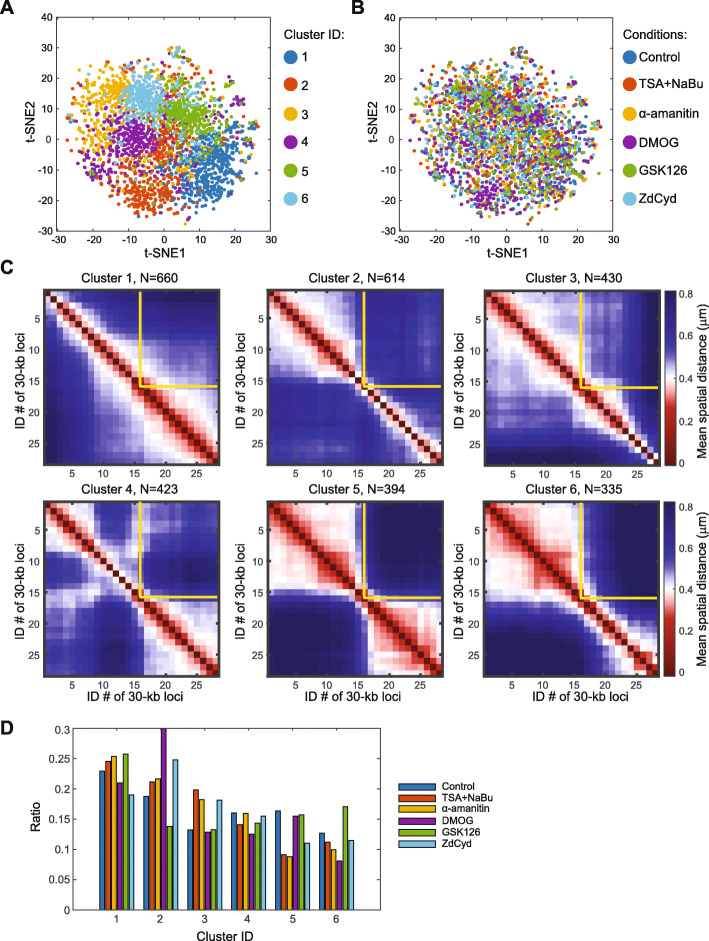
Clusters of chromatin conformations under perturbations of epigenetic components and transcription machinery (target region as stated in Fig. 1). **A** Louvain-Jaccard clustering result of chromatin traces displayed with t-distributed stochastic neighbor embedding (t-SNE). Each dot represents a chromatin trace. Different pseudo-colors represent different chromatin trace clusters. **B** The same t-SNE plot of chromatin traces as in **A** but colored by different perturbation conditions. **C** Mean spatial distance matrices for the six chromatin trace clusters identified in **A**. The yellow lines represent the ensemble TAD boundary. **D** Ratio of chromatin trace copy number in each cluster over that in all clusters for each perturbation (and control). *N* = 2856 for **A**, **B**, and **D**

To further resolve the influences of different epigenetic perturbations on chromatin conformations, we combined chromatin traces from all epigenetic perturbation conditions and control and performed Louvain-Jaccard clustering [49, 50] of the traces as in Additional file 1: Fig. S1. A total of six conformational clusters were revealed (Fig. 4A). The clusters do not correspond to different epigenetic perturbations, as chromatin traces from different perturbations and control were highly dispersed among the clusters (Fig. 4B). Mean spatial distance matrices of the clusters showed distinct chromatin conformations (Fig. 4C). Different epigenetic perturbations and control were roughly equally represented in each cluster (Fig. 4D). And each cluster contained a mixture of Xi and Xa traces (Additional file 1: S10). Taken together, these analyses indicated that the perturbations of several major epigenetic components and transcription did not systematically alter the diverse chromatin conformations of the traced X chromosome region.

**Fig. 5:**
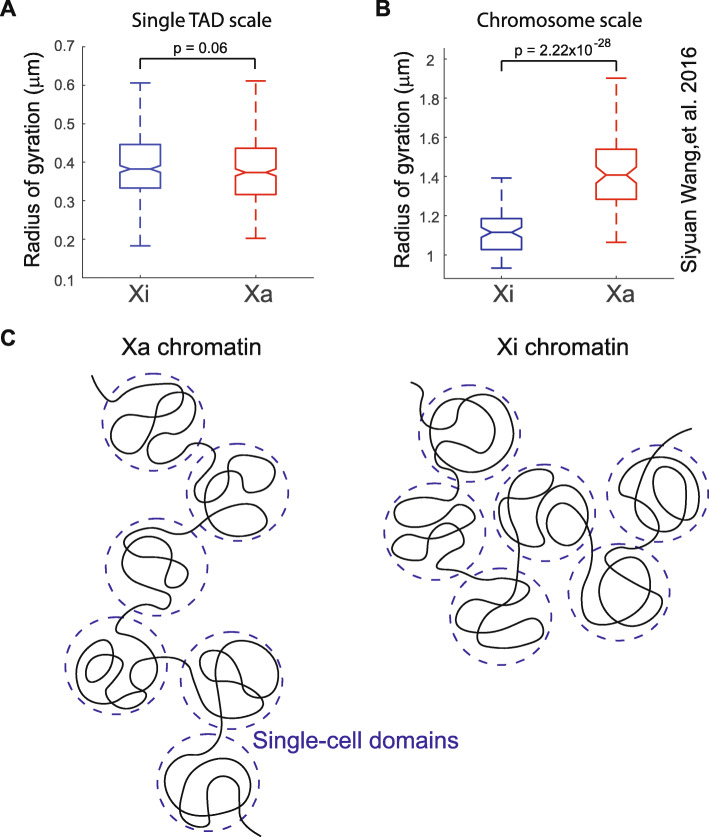
Increased compaction of inactive X chromosomes in comparison to active X chromosomes is present at the TAD-to-chromosome scale, but not at the TAD/sub-TAD scale. **A** Radii of gyration of inactive (left, *N* = 403) versus active X chromosomes (right, *N* = 421) from fine-scale chromatin tracing of the 840-kb region (target region of **A** as stated in Fig. 1). **B** Radii of gyration of inactive (left, *N* = 95) versus active X chromosomes (right, *N* = 95) from large-scale chromatin tracing of 40 TADs across the entire X chromosome. **C** Schematic illustration of non-proportional compaction of inactive X chromosomes at length scales above the TADs/single-cell domains. The *p* values were calculated for radii of gyration using two-sided Student’s *t* tests. Boxes are defined as in Fig. 3G

Finally, we asked if the increased level of chromatin compaction in Xi in comparison to Xa is present at the scale of single-cell domains. Xi is known to be more spatially compact than Xa at the whole chromosome level [51]. This does not require/imply the compaction to be present/proportional at all length scales. To identify the length scale at which this compaction occurs, we calculated the radii of gyration of the traced genomic regions in Xi and Xa. The radius of gyration of a trace reflects the radius of the spatial volume spanned by the trace’s folding conformation. The comparison showed that in all three traced genomic regions, the Xa and Xi traces had similar radii of gyration at the length scale of single TADs (Fig. 5A, Additional file 1: S11A-B). In fact, in the two open chromatin regions among the three regions (the first traced region is largely an open chromatin region as shown in Additional file 1: Fig. S2A), the Xa traces appear to have slightly smaller radii of gyration than Xi traces (Fig. 5A, Additional file 1: S11A). However, when larger scale chromosome traces from our previous study [30] were analyzed in the same fashion, the analyses showed that Xa had significantly larger radii of gyration than Xi at the TAD-to-chromosome length scale (Fig. 5B). These results indicate that the chromatin compaction associated with X inactivation occurs at a length scale above that of TADs and single-cell domains (Fig. 5C).

## Discussion

Loop extrusion and epigenetic landscape have been shown to be major, parallel contributing factors to the spatial organization of TADs [26, 27]. As the disruption of loop extrusion did not abolish single-cell domains [32], recent studies proposed that epigenetic interactions mediate the formation of single-cell domains and demonstrated such possibility with computational modeling [34, 35]. To recapitulate the highly variant single-cell domains, the modeling involves several constant epigenetic profiles (termed “binding sites” in the model) across a genomic region; different epigenetic reader proteins (termed “binders” in the model) dynamically bind to the corresponding epigenetic binding sites and can mediate the interactions between pairs of genomic loci with the same kind of epigenetic binding sites; the thermodynamics of the chromatin polymer leads to contacts between genomic loci, some of which are captured by the epigenetic interactions mediated by the binders; due to the thermodynamic nature of the system, the distribution of the chromatin contact instances and the epigenetic interaction instances differ from one copy of the polymer to another, which underlies the different single-cell domain structures [34, 35]. In this work, we showed that ensemble TADs on the inactive X chromosome were attenuated (but not completely lost) whereas TADs on the active X chromosome were prominent, consistent with previous findings in mouse [44, 52, 53]. Strikingly, the active and inactive X chromosomes, despite having distinct epigenetic states [36, 38, 54], both can contain single-cell domains of similar features. This observation did not appear to depend on a particular human cell type or on the open or closed states of the chromatin region. A concurrent preprint also reported the observation of single-cell domains on both Xa and Xi chromosomes in mouse brain tissue [55], supporting the prevalence of these structures. We further showed that several perturbations of major epigenetic and transcription components, including histone acetylation and methylation, DNA methylation, and RNA polymerase, did not abolish the single-cell domains. These results did not support the model in which epigenetic interactions mediate single-cell domain formation. In addition, we showed that the chromatin compaction associated with X inactivation occurs at a length scale above that of TADs and single-cell domains.

Due to the limitation of this study, we cannot rule out the possibility that some untested epigenetic components, including various histone and DNA modifications and non-histone DNA binding proteins, underlie the formation of single-cell domains. Neither can we rule out the possibility that the tested epigenetic components are compensated by other epigenetic components. In addition, Xa and Xi-specific allelic profiles of the tested epigenetic components in the IMR-90 cell line are lacking. Whether our observations can be generalized to the entire X chromosome or to the whole genome is also unknown. Thus, this work does not disprove the epigenetic model. However, our data suggest that alternative explanations for the formation of single-cell domains may deserve equal consideration. For example, an unknown motor protein other than cohesin may drive the formation of single-cell domains through loop extrusion. Alternatively, some nuclear components may form scaffolds or aggregates that push away chromatin into adjacent pockets of free nuclear space that correspond to single-cell domains. Further studies may reveal the mechanism underlying the single-cell domains, as well as their dynamics and functional significance in single cells. We also note the recent reports of multiple highly variant chromatin domain structures at the single-cell level observed with different advanced technologies, e.g., nucleosome clutches [7], chromatin nanodomains (CNDs) [20], packing domains (PDs) [56], and larger single-cell domains (SCDs) that are multi-megabase in size [57]. The relationship between these structures and the TAD-like single-cell domains identified by chromatin tracing also requires further investigation.

## Conclusions

We conclude that TAD-like structures/single-cell domains exist on both active and inactive X chromosomes and persist under perturbations of major epigenetic components and transcription. These observations point towards a mechanism that does not rely on cohesin or major epigenetic marks for the establishment/maintenance of single-cell domains, an apparently prevalent building block of genome architecture.

## Methods

### Probe design and synthesis

To design DNA FISH probes for chromatin tracing, the main target genomic region of interest (ChrX: 76,800,000–77,640,000, hg18) and the additional closed (ChrX: 8,280,000–9,120,000, hg18) and open (ChrX: 100,040,000–100,880,000, hg18) chromatin regions were each divided into 28 consecutive 30-kb target segments. To design probes for the main target genomic region of interest (ChrX: 76,800,000–77,640,000, hg18), for each 30-kb target segment, 400 oligonucleotides were designed as template oligos. On each template oligo, the following sequences were concatenated from 5′ to 3′: (1) a 20-nucleotide (nt) forward priming sequence, (2) a 30-nt secondary probe binding sequence, (3) a 30-nt genome targeting sequence, and (4) a 20-nt reverse priming sequence. The 30-nt genome targeting sequences were designed with the software OligoArray 2.1 [58] using the following criteria: The melting temperatures of the targeting sequences are between 60 and 100 °C; the melting temperatures of potential secondary structures in the sequences do not exceed 70 °C; the melting temperatures of potential cross hybridization among the sequences do not exceed 70 °C; the GC contents of the targeting sequences are between 30 and 90%; there are no consecutive repeats of seven or more A’s, G’s, C’s, and T’s; the targeting sequences do not overlap. The chosen targeting sequences were further checked with BLAST+ [59] and only sequences that appear once in the human genome were retained. To design probes for the additional closed (ChrX: 8,280,000–9,120,000, hg18) and open (ChrX: 100,040,000–100,880,000, hg18) chromatin regions, we designed 400 template oligonucleotides for each 30-kb target segment. Each template oligo contains (from 5′ to 3′): (1) a 20-nt forward priming sequence, (2) a 20-nt secondary probe binding sequence, (3) a 30-nt genome targeting sequence, (4) a same 20-nt secondary probe binding sequence, and (5) a 20-nt reverse priming sequence. The 30-nt genome targeting sequences were designed with ProbeDealer [60]. The secondary probe binding sequences and priming sequences were introduced in previous studies [30, 43]. The genomic coordinates of the targeting regions are provided in Additional file 2: Supplementary Table 1. The template oligo pool sequences were provided in Additional file 3: Supplementary Table 2.

To design *XIST* RNA FISH probes, we downloaded the *XIST* transcript sequences from https://genome.ucsc.edu/cgi-bin/hgGene?hgg_gene=uc004ebm.2&hgg_prot=uc004ebm.2&hgg_chrom=chrX&hgg_start=73040485&hgg_end=73072588&hgg_type=knownGene&db=hg19&hgsid=666371357_9PdT3XyjXSVOpavdVHxoox2IVEBf. We then designed 50 template oligos targeting the transcript. Each template oligo contained (from 5′ to 3′) a 15-nt forward priming sequence, a 30-nt transcript targeting sequence, and a 15-nt reverse priming sequence. The 15-nt priming sequences were truncated from previously used priming sequences [30, 43]. The 30-nt transcript targeting sequences were designed using the OligoArray 2.1 [58] software with the following parameters: The melting temperatures of the targeting sequences are between 66 and 100 °C; the melting temperatures of potential secondary structures in the sequences do not exceed 76 °C; the melting temperatures of potential cross hybridizations among the sequences do not exceed 72 °C; the GC contents of the targeting sequences are between 30 and 90%; there are no consecutive repeats of six or more G’s, C’s, T’s, and A’s; the targeting sequences do not overlap. We further checked the sequences of the template oligos with BLAST+ [59] and only retained sequences that appeared once in the human genome. The template oligo pool sequences for *XIST* RNA FISH probes are provided in Additional file 4: Supplementary Table 3.

To design intron RNA FISH probes for genes *TMSB4X* and *RPL36A*, we downloaded intron transcript sequences of the genes from UCSC genome browser, and designed template oligo pools for the intron RNA FISH using ProbeDealer [60]. A total of 24 template oligos were designed for *TMSB4X* and 45 template oligos were designed for *RPL36A*. On each template oligo, the following sequences were concatenated from 5′ to 3′: (1) a 20-nt forward priming sequence, (2) a 20-nt secondary probe binding sequence, (3) a 30-nt intron targeting sequence, (4) a same 20-nt secondary probe binding sequence, and (5) a 20-nt reverse priming sequence. The template oligo pool sequences for *TMSB4X* and *RPL36A* intron RNA FISH probes are provided in Additional file 5: Supplementary Table 4.

The chromatin tracing and intron RNA FISH template oligo pool was ordered from CustomArray, GenScript. The *XIST* RNA FISH template oligo pool was ordered from Integrated DNA Technologies (IDT), Inc. Primary probes were synthesized from the template oligo pool via limited-cycle PCR, in vitro transcription, reverse transcription, alkaline hydrolysis, and probe purification [30, 61, 62]. All PCR primers and dye-labeled reverse transcription primers used in probe synthesis, as well as dye-labeled secondary probes used in the “Secondary probe sequential hybridization” section, were purchased from Integrated DNA Technologies (IDT), Inc. We used Alexa Fluor 647-labeled reverse transcription primers to synthesize chromatin tracing primary probes for datasets collected via “Combined chromatin tracing primary hybridization with mH2A1 immunofluorescence staining” so that all primary probes were labeled with Alexa Fluor 647 fluorophores. We used Cy5-labeled and ATTO 565-labeled reverse transcription primers to synthesize *XIST* RNA FISH probes and chromatin tracing primary probes respectively for datasets collected via “Combined chromatin tracing primary hybridization with *XIST* RNA FISH”. We used unlabeled reverse transcription primers to synthesize intron RNA FISH probes. The sequences of the primers were listed in Additional file 6: Supplementary Table 5.

### Cell culture

The IMR-90 cell line and hTERT-RPE-1 cell line were purchased from American Type Culture Collection (ATCC, cat. no. CCL-186 for IMR-90 and cat. no. CRL-4000 for hTERT-RPE-1). IMR-90 cells were cultured with Eagle’s MEM (ATCC, cat. no. 30-2003) containing 10% (vol/vol) FBS and 1× penicillin-streptomycin. The hTERT-RPE-1 cells were cultured with DMEM: F12 (ATCC, cat.no. 30-2006) medium containing 10% (vol/vol) FBS and 1× penicillin-streptomycin. For imaging, cells were plated onto 40-mm-diameter, #1.5 coverslips in Falcon 60-mm tissue culture dishes. When the cells reached a confluency of 70%, the cells were fixed with 4% (wt/vol) paraformaldehyde (PFA) in DPBS for 10 min at room temperature and washed twice with Dulbecco’s phosphate-buffered saline (DPBS). The cell lines were not authenticated or tested for mycoplasma contamination.

### Drug treatment

To inhibit transcription, IMR-90 cells were cultured in cell media supplemented with 50 μg/mL α-Amanitin (Sigma-Aldrich, cat.no. A2263) for 8 h before PFA fixation. To inhibit HDAC function, IMR-90 cells were cultured in cell media supplemented with 100 ng/mL TSA (Sigma-Aldrich, cat.no. T8552) plus 20 mM NaBu (Sigma-Aldrich, cat.no. B5887) for 24 h before PFA fixation. To inhibitor EZH2 methyltransferase, IMR-90 cells were cultured in cell media supplemented with 1 μM GSK126 (Sigma-Aldrich, cat. no. 5.00580) for 48 h before PFA fixation. To induce hyper histone methylation, IMR-90 cells were cultured in cell media supplemented with 2.5 mM DMOG (Sigma-Aldrich, cat.no. D3695) for 12 h before PFA fixation. To inhibit DNA methylation (cytosine-C5), IMR-90 cells were cultured in cell media supplemented with 10 μM ZdCyd (Sigma-Aldrich, cat.no. 189825) for 24 h before PFA fixation.

### Combined chromatin tracing primary hybridization with mH2A1 immunofluorescence staining

For the main target region (ChrX: 76,800,000–77,640,000, hg18), half of the untreated IMR-90 datasets and all hTERT-RPE-1 datasets were collected with “Combined chromatin tracing primary hybridization with mH2A1 immunofluorescence staining”. The fixed and washed IMR-90 cells or hTERT-RPE-1 cells were treated with freshly made 1 mg/mL sodium borohydride in DPBS for 10 min at room temperature, and washed twice with DPBS for 2 min each. The cells were then permeabilized with 0.5% (vol/vol) Triton X-100 in DPBS for 10 min at room temperature and washed twice with DPBS for 2 min each. Then the cells were treated with 0.1 M HCl for 5 min at room temperature and washed twice with DPBS. Next the cells were treated with freshly prepared 0.1 mg/mL ribonuclease A solution for 45 min at 37 °C. The cells were then washed with 2× saline-sodium citrate (SSC) buffer twice and incubated at room temperature for 30 min in pre-hybridization buffer composed of 50% (vol/vol) formamide and 0.1% (vol/vol) Tween-20 in 2× SSC. Hybridization buffer composed of 50% (vol/vol) formamide and 20% (wt/vol) dextran sulfate in 2× SSC containing 6–20 μM chromatin tracing primary probes was pipetted onto a glass slide. The cell coverslip was flipped onto the glass slide so that cells were in contact with the hybridization buffer. The coverslip-slide assembly was placed onto an 86 °C digital dry bath and denatured for 3 min. The assembly was incubated overnight for 15–18 h at 37 °C in a humid chamber. The cells were then washed with 2× SSCT (2× SSC and 0.1% (vol/vol) Tween-20) at 60 °C in a water bath for 15 min twice. The cells were further washed with 2× SSCT at room temperature for 15 min. Next the cells were blocked with 3% (wt/vol) BSA (Sigma-Aldrich, cat. no. A9647-100G) and 0.2% (vol/vol) Triton X-100 in DPBS for 30 min. The cells were then incubated with rabbit mH2A1 primary antibodies (Abcam, ab183041) at a concentration of 1:100 in antibody dilution buffer containing 1% (wt/vol) BSA and 0.2% (vol/vol) Triton X-100 for 1 h at room temperature, and washed three times for 5 min each in DPBS containing 0.05% (vol/vol) Triton X-100. The cells were then incubated with Alexa Fluor 568-labeled goat anti-rabbit secondary antibodies (Invitrogen, A-11011) in antibody dilution buffer for 1 h at room temperature and washed three times for 5 min each in DPBS containing 0.05% (vol/vol) Triton X-100. Then 0.1-μm yellow-green fiducial beads (Invitrogen, F8803) were resuspended in 2× SSC and applied to the cells so that they could serve as fiducial markers to cancel the sample drift during the sequential hybridization.

### Combined chromatin tracing primary hybridization with *XIST* RNA FISH

For the main target region (ChrX: 76,800,000–77,640,000, hg18), half of the untreated IMR-90 datasets and all drug-treated IMR-90 datasets were collected with “Combined chromatin tracing primary hybridization with *XIST* RNA FISH”. Datasets of the two additional closed and open regions (ChrX: 8,280,000–9,120,000, hg18 and ChrX: 100,040,000–100,880,000, hg18) from untreated IMR-90 cells were also collected with “Combined chromatin tracing primary hybridization with *XIST* RNA FISH”. The fixed and washed IMR-90 cells were permeabilized with 0.5% (vol/vol) Triton X-100 in DPBS for 10 min at room temperature and washed twice with DPBS for 2 min each. The cells were then treated with 0.1 M HCl for 5 min at room temperature and washed twice with DPBS. Next, the cells were incubated at room temperature for 30 min in pre-hybridization buffer composed of 50% (vol/vol) formamide and 2 mM Ribonucleoside vanadyl complexes (Sigma-Aldrich, R3380) in 2× SSC buffer. Hybridization buffer composed of 50% (vol/vol) formamide, 0.1% (wt/vol) yeast tRNA (Life Technologies, 15401011), 10% (wt/vol) dextran sulfate (Sigma, D8906-50G), and 100× diluted murine RNase inhibitor in 2× SSC was pipetted onto a glass slide. The coverslip was flipped onto the glass slide so that cells were in contact with the hybridization buffer. The coverslip-slide assembly was placed onto an 86 °C digital dry bath and denatured for 3 min. The coverslip was then removed and briefly washed with 2× SSC. The sample was then incubated with hybridization buffer containing 6–20 μM chromatin tracing primary probes and 1 μM XIST RNA FISH probes overnight for 15–18 h at 37 °C in a humid chamber. The cells were then washed with 2× SSCT at 60 °C in a water bath for 15 min twice and further washed with 2× SSCT for 15 min and 2× SSC for 3 min at room temperature. The 0.1-μm yellow-green fiducial beads (Invitrogen, F8803) were resuspended in 2× SSC and applied to the cells so that they could serve as fiducial markers to cancel the sample drift during the sequential hybridization.

### Secondary probe sequential hybridization

After the primary probe hybridization, the sample was assembled into a Bioptech’s FCS2 flow chamber and repetitively hybridized with Alexa Fluor 647 and ATTO 565-labeled secondary probes (Additional file 6: Supplementary Table 5), imaged and photobleached.

To perform buffer exchange automatically during the secondary probe sequential hybridization procedure, we used a computer-controlled, home-built fluidics system [30, 61]. Prior to the secondary probe sequential hybridization, in a first imaging round denoted as hybridization round 0 (hyb 0), for datasets collected with “Combined chromatin tracing primary hybridization with mH2A1 immunofluorescence staining”, we imaged the mH2A1 immunofluorescence patterns and imaged the entire targeted chromatin region with z-stepping in the 560-nm channel and 647-nm channel with the Dual-View setup respectively in 2× SSC. For datasets collected with “Combined chromatin tracing primary hybridization with *XIST* RNA FISH”, we imaged the *XIST* RNA FISH signals and visualized the targeted chromatin region with z-stepping in the 647-nm and 560-nm channels respectively in oxygen scavenging imaging buffer [63] (50 mM Tris-HCl pH 8.0, 10% (wt/vol) glucose, 2 mM Trolox (Sigma-Aldrich, 238813), 0.5 mg/mL glucose oxidase (Sigma-Aldrich, G2133), 40 μg/mL catalase (Sigma-Aldrich, C30), 0.05% (vol/vol) murine RNase inhibitor in 2× SSC). We covered the oxygen scavenging imaging buffer under a layer of mineral oil (Sigma, 330779) to prevent continuous oxidation. After hyb 0 imaging, we photobleached all the signals in hyb 0. For each round of the secondary probe hybridization, the sample was first hybridized with 7.5 nM each of the Alexa Fluor 647 and ATTO 565-labeled secondary probes in 20% (vol/vol) ethylene carbonate (Sigma-Aldrich, E26258) in 2× SSC and incubated at room temperature for 20 min. We then sequentially flowed through the chamber 2 mL wash buffer containing 20% (vol/vol) ethylene carbonate in 2× SSC and 2 mL imaging buffer (either 2× SSC for “Combined chromatin tracing primary hybridization with mH2A1 immunofluorescence staining” experiments or the oxygen scavenging imaging buffer [63] in “Combined chromatin tracing primary hybridization with *XIST* RNA FISH” experiments).

Next, we took z-stepping images with 647-nm, 560-nm, and 488-nm laser illuminations for Alexa Fluor 647 and ATTO 565-labeled secondary probes and fiducial beads respectively, with 200-nm step sizes and 0.4-s exposure time at each step. The z-stacks cover a range of 7 μm in z. After the imaging, we switched the sample buffer to 2× SSC and performed photobleaching with simultaneous 647-nm and 560-nm laser illuminations for 25 s. We performed 14 rounds of secondary hybridization (hyb) in total. The first 14 regions were imaged in the 647-nm channel from hyb 1 to hyb 14 and the last 14 regions were imaged in the 560-nm channel from hyb 1 to hyb 14.

### DNA FISH of main target region with co-immunofluorescence staining for epigenetics marks

The 4% PFA fixed IMR-90 cells were permeabilized with 0.5% (vol/vol) Triton X-100 in DPBS for 15 min at room temperature and washed twice with DPBS for 2 min each. Next, the cells were blocked with blocking buffer comprised of 3% (wt/vol) BSA and 0.2% (vol/vol) Triton X-100 in DPBS for 30 min. Then, the cells were incubated with 1:100 diluted H3K27me3 (Abcam Cat# ab6002) or H3K27ac antibody (Abcam Cat# ab4729) in blocking buffer for 1 h at room temperature. After three times of DPBS wash for 5 min each, cells were incubated with 1:1000 diluted Alexa Fluor 647 labeled secondary antibody (Invitrogen, A21237 for H3K27me3 staining or Invitrogen, A31573 for H3K27ac staining) in blocking buffer for 1 h at room temperature. Cells were then washed three times with DPBS for 5 min each. To post-fix the antibody signal, cells were first incubated with freshly prepared 4% PFA in 1× DPBS at room temperature for 5 min and then washed with 1× DPBS six times at room temperature for 15 min in total. Cells were further fixed with 1.5 mM PEGylated bis(sulfosuccinimidyl)suberate (BS(PEG)5) (Thermo Scientific, 21581) in 1× DPBS for 20 min at room temperature. The reaction was quenched by incubating with 100 mM Tris-HCl pH 7.4 for 5 min at room temperature. Then, the cells were processed with standard DNA FISH protocol with ATTO 565-labeled primary FISH probes targeting the main target region (ChrX: 76,800,000–77,640,000, hg18), as described in the “Combined chromatin tracing primary hybridization with *XIST* RNA FISH” section. Finally, the cells were incubated with diamidino-2-phenylindole (DAPI; Thermo Scientific, 62248) in DPBS at a 1:1000 concentration for 10 min at room temperature and washed twice with 1× DPBS at room temperature for 3 min each. Immunofluorescence staining patterns were imaged with 638-nm laser illumination. DNA FISH signals were imaged with 546-nm laser illumination. DAPI nucleus staining signals were imaged with the 405-nm laser illumination. We took z-stepping images to cover the nucleus with 200 nm step sizes and 0.4 s exposure time at each step.

### Intron FISH

The fixed and washed IMR-90 cells were first permeabilized with 0.5% (vol/vol) Triton X-100 in DPBS for 15 min at room temperature and washed twice with DPBS for 2 min each. Then, the cells were incubated in pre-hybridization buffer composed of 50% (vol/vol) formamide, 2 mM Ribonucleoside vanadyl complexes, and 1000x diluted murine RNase inhibitor in 2× SSC for 15 min at room temperature. A total of 25 μL of hybridization buffer composed of 50% (vol/vol) formamide, 0.1% (wt/vol) yeast tRNA, 10% (wt/vol) dextran sulfate, 100× diluted murine RNase inhibitor and 6 μM intron FISH primary probes in 2× SSC were added on a parafilm. The cell coverslip was flipped onto the hybridization buffer and incubated overnight for 24 h at 37 °C in a humid chamber. On the next day, the cells were washed with 2× SSCT at 60 °C in a water bath for 15 min twice and further washed with 2× SSCT for 15 min and 2× SSC for 3 min at room temperature. Next, samples were incubated with 3.25 nM dye-labeled secondary probes in 20% (vol/vol) ethylene carbonate in 2× SSC and at room temperature for 20 min. We used an Alexa Fluor 647-labeled secondary probe (Additional file 6: Supplementary Table 5, FB1) for *TMSB4X* and an Alexa Fluor 750-labeled secondary probe (Additional file 6: Supplementary Table 5, FB2) for *RPL36A.* Then, samples were washed with wash buffer containing 20% (vol/vol) ethylene carbonate in 2× SSC and 2× SSC sequentially for 10 min each at room temperature. Intron RNA FISH signals for *TMSB4X* and *RPL36A* were imaged with 638-nm and 749-nm laser illuminations, respectively. We took z-stepping images to cover the nucleus with 200 nm step sizes and 0.4 s exposure time at each step.

### Time-course immunofluorescence imaging

All the time-course immunofluorescence staining experiments were performed in the following steps except for 5-methylcytosine (5-mC) staining. The fixed and washed IMR-90 cells after various lengths of drug treatments were first permeabilized with 0.5% (vol/vol) Triton X-100 in DPBS for 15 min at room temperature and washed twice with DPBS for 2 min each. The cells were then blocked in the blocking buffer for 30 min at room temperature. Then, the cells were incubated in 1:100 diluted antibody (Abcam Cat# ab4729 for H3K27ac staining, Abcam Cat# ab8898 for H3K9me3 staining, Abcam Cat# ab6002 for H3K27me3 staining) for 1 h at room temperature. After three times of DPBS wash for 5 min each, cells were incubated with 1:1000 diluted Alexa Fluor 647 labeled secondary antibody (Invitrogen, A31573 for H3K27ac and H3K9me3 staining, Invitrogen, A21237 for H3K27me3 staining) for 1 h at room temperature. Cells were further washed three times with DPBS for 5 min each. As for 5-mC staining, cells were incubated with freshly made 2 M hydrochloric (HCl) acid in H_2_O at 37 °C in a humid chamber after permeabilization. After HCl denaturation, cells were neutralized by incubation in 0.1 M Tris-HCl (pH 8.0) for 10 min at room temperature. Then, the cells were processed with blocking and antibody incubation as described above except with anti-5-mC antibodies (Invitrogen, MA5-24694). Finally, DAPI staining was performed by incubating with 1:1000 diluted DAPI in DPBS for 10 min at room temperature and washed twice with 1× DPBS at room temperature for 3 min each. Immunofluorescence staining patterns were imaged with 638-nm laser illumination. DAPI nucleus staining signals were imaged with the 405-nm laser illumination. We took z-stepping images to cover the nucleus with 200-nm step sizes and 0.4-s exposure time at each step.

### Image system

We used a home-built microscope system with a Nikon Ti2-U body equipped with a Nikon CFI Plan Apo Lambda 60× Oil (numerical aperture: 1.40) objective lens, an active auto-focusing system, and a Hamamatsu Orca Flash 4.0 V3 camera. The pixel size of our system was 107.9 nm. For epifluorescence illumination, we used a 750-nm laser (2RU-VFL-P-500-750-B1R, MPB Communications) to excite and image Alexa Fluor 750, a 647-nm laser (2RU-VFL-P-1000-647-B1R, MPB Communications) to excite and image Cy5 and Alexa Fluor 647 fluorophores, a 560-nm laser (2RU-VFL-P-1000-560-B1R, MPB Communications) to excite and image ATTO 565 and Alexa Fluor 568 fluorophores, and a 488-nm laser (2RU-VFL-P-500-488-B1R, MPB Communications) to excite and image the yellow-green fiducial beads for drift correction. The microscope body hosts a penta-band dichroic mirror (ZT405/488/561/647/752rpc-UF2, Chroma), a penta-band emission filter (Chroma, ZET405/488/561/647-656/752 m), a motorized x–y sample stage (SCAN IM 112×74, Marzhauser), a piezo z positioner (Mad City Labs, Nano-F100S), and a home-built focus-lock system as described previously [41]. For datasets collected with “Combined chromatin tracing primary hybridization with mH2A1 immunofluorescence staining”, we installed a Duel-View setup on the emission path to prevent the fluorescent bleed-through between the mH2A1 and chromatin imaging channels. The Dual-View setup included a 690-nm long-pass dichroic mirror to isolate the emission wavelengths, an FF01-607/70 (Semrock) bandpass filter for fluorescence from the 488-nm and 560-nm channels, and an ET720/60 m (Chroma) bandpass filter for fluorescence emissions in the 647-nm channel. To align the 560-nm and 647-nm Duel-View channels and to cancel the color shift, we took z-stack calibration images of 100-nm TetraSpeck beads (Invitrogen). To control the laser intensities of the three lasers above, we used either a filter wheel with neutral density filters (FW102C, Thorlabs) or an acousto-optic tunable filter (AOTF, 97-03309-01 Gooch & Housego) to tune the laser intensities during imaging, and four mechanical shutters (LS3S2Z0, Vincent Associates) to control laser on-off.

For some experiments described in the “DNA FISH of main target region with co-immunofluorescence staining for epigenetics marks” section and “Time-course immunofluorescence imaging” section, we used a similar image system with the same microscope body, objective lens, camera, stage, piezo z positioner and focus lock as above, but with a Lumencor CELESTA fiber-coupled solid-state laser with five lines at 405-nm, 477-nm, 546-nm, 638-nm, and 749-nm, and a corresponding set of penta-band dichroic mirror and emission filter from Lumencor. The 638-nm line was used to image Alexa Fluor 647 dye. The 546-nm line was used to image ATTO 565 dye. The 405-nm line was used to image DAPI.

### Data analysis

#### Classification of Xa and Xi

For datasets with strong *XIST* FISH signals, we quantified the *XIST* FISH intensity values at the positions of the chromatin trace region in hyb 0 and plotted the distributions of the intensity values. The distribution pattern is typically bimodal due to the enrichment of *XIST* on Xi. We then used the intensity values that separate the two peaks in the distribution as the threshold to classify Xa and Xi. For datasets with mH2A1 immunofluorescence or relatively weak *XIST* FISH signals, to ensure the accuracy of the Xa-versus-Xi classification, we manually selected Xa and Xi traces based on the absence or presence of mH2A1/*XIST* co-localization with the chromatin trace region in hyb 0. We only analyzed cells with two distinct chromatin trace regions in hyb 0 to avoid replicating or non-diploid cells.

#### Reconstruction of 3D chromatin traces

To reconstruct the 3D chromatin traces, we first corrected the color shift between the 560-nm and 647-nm laser channels with TetraSpeck bead images and measured the sample drift during sequential hybridization and imaging with fiducial bead images. We identified fluorescence loci by local intensity thresholding and measured the 3D positions of each locus with 3D Gaussian fitting in the *x*, *y*, *z* dimensions. The drift-corrected loci were then linked into chromatin traces based on their spatial clustering patterns. If the chromatin traces have missing loci after the initial loci fitting and linking, we attempted to re-fit the 3D positions of the missing loci within the chromatin trace region in corresponding hybridization rounds with 3D Gaussian fitting. The re-fitted loci were then added to the chromatin traces.

#### Hi-C data analysis

We downloaded Hi-C data of IMR-90 cells from Gene Expression Omnibus GSE63525 [16]. We first binned the Hi-C contact counts of 5-kb resolution into 30-kb bins, which is the resolution of the chromatin tracing, and summed all contact counts between each pair of traced regions. We defined the summed value as the Hi-C number of reads or contact frequencies.

#### CTCF and RAD21 binding analyses

CTCF and RAD21 binding sites were directly retrieved from narrow peak calls analyzed from ChIP-seq data for IMR-90 cell lines [42]. The narrow peak binding sites were called by the ENCODE Consortium [42] and downloaded from the following UCSC website. CTCF binding in IMR-90: http://hgdownload.cse.ucsc.edu/goldenPath/hg19/encodeDCC/wgEncodeAwgTfbsUnifor m/wgEncodeAwgTfbsSydhImr90CtcfbIggrabUniPk.narrowPeak.gz. RAD21 binding in IMR-90: http://hgdownload.cse.ucsc.edu/goldenPath/hg19/encodeDCC/wgEncodeAwgTfbsUnifor m/wgEncodeAwgTfbsSydhImr90Rad21IggrabUniPk.narrowPeak.gz.

#### ATAC-seq, ChIP-seq, and GRO-seq data analyses

We downloaded the ATAC-seq call sets of IMR-90 cells from the ENCODE portal (https://www.encodeproject.org/) with the identifier ENCSR200OML [42, 64]. We downloaded the ChIP-seq call sets of IMR-90 cells from the ENCODE portal with the following identifiers: H3K9me3 (ENCSR055ZZY), H3K4me3 (ENCSR087PFU), H3K27me3 (ENCSR431UUY), H3K27ac (ENCSR002YRE), and H3K9ac (ENCSR219MYH) [42, 64]. We downloaded the GRO-seq dataset from a previous publication [65].

#### Boundary strength, boundary probability, and boundary frequency analyses

Chromatin traces with no less than 23 loci were used for boundary strength, boundary probability, and boundary frequency calculations. For each chromatin trace, we reconstructed a spatial distance matrix, with each element representing the Euclidean distance between each pair of traced loci. Missing values were interpolated using linear interpolations of neighboring, non-missing values. To measure the boundary strengths for each locus, we calculated the median values (L) of the left three columns excluding the locus, each extending 10 elements below the diagonal, the median values (R) of the right three 10-element columns including the locus below the diagonal, the median values (T) of the left three 10-element columns including the locus above the diagonal, and the median values (B) of the right three 10-element columns excluding the locus above the diagonal. We then defined two boundary strengths: start-of-domain boundary strength (L/R) and end-of-domain boundary strength (B/T). Using the start-of-domain and end-of-domain boundary strengths, we identified start/end boundary positions by calling the local maximum above a defined threshold. Based on the boundary positions, we computed the start/end boundary probability for each locus as the fraction of chromosomes in which the corresponding locus is identified as a boundary. The average of the start and end boundary probabilities for each locus is defined as the boundary probability of that locus. For each group of chromatin traces, we defined the boundary frequency as the average number of boundaries per trace.

#### Conditional and unconditional probability analyses

To perform conditional and unconditional probability analyses, we first ordered all triplets of chromatin segments within the traced genomic region. Chromatin segments within a distance threshold of 200-nm are defined as in contact with each other. For each triplet of segments A, B, and C (ordered from 5′ to 3′ of the traced region), we define the following three probabilities. The unconditional probability between B and C is defined as the percentage of traces with B-C contact among all traces with detected B and C. The conditional probability of B-C contact given A-B contact is defined as the percentage of traces with B-C contact among traces with A-B contact. In this case, we first isolated all traces with A-B contact and calculated the probability of B-C contact among these traces. The conditional probability of B-C contact given no A-B contact is defined as the percentage of traces with B-C contact among traces without A-B contact. In this case, we first isolated all traces without A-B contact and calculated the probability of B-C contact among these traces.

#### Quantification for antibody staining and intron FISH signals

To quantify the antibody signal in IF experiments, the cell nuclei were first segmented using DAPI staining (for Additional file 1: Fig S6 C, E) or nucleus enriched antibody signal (Additional file 1: Fig S6 A-B). The DAPI or antibody staining z-stepping images were first averaged along the *z*-axis to generate an average *z*-projection image. Then, a background image was generated using adaptthresh function in MATLAB with a large neighborhood size. We removed the background by division and normalized the image. Next, image closing and opening by reconstruction were performed sequentially with a disk-shaped morphological structuring element. Then, we generated the regional maxima image from the reconstructed image and further filled the holes in the regional maxima image. An iterative region-growing image segmentation algorithm (activecontour function in MATLAB) was applied using the reconstructed image and regional maxima image, and the cell nucleus shapes were segmented. Some misidentified cell nuclei were removed by manual selection. Finally, the antibody staining image was overlaid on the nuclear segmentation and the averaged antibody signal intensity was calculated in each cell nucleus. To quantify the antibody signal for combined DNA FISH and co-IF experiments, we first fitted the 3D position of DNA foci in each field of view. Then, the antibody signal intensity at each fitted position was extracted. The intron FISH foci numbers per cell nucleus were derived by manual counting.

#### Radius of gyration calculation

To calculate the radius of gyration for each chromatin trace, we measured the sum squared Euclidean distance between each traced locus to the centroid of the chromatin trace and normalized it to the number of traced loci. The radius of gyration is defined as the square-root of the normalized value, as in the formula below. In the formula, xi, yi, and zi represent the *x*, *y*, *z* coordinates of one target locus; xc, yc, and zc represent the *x*, *y*, *z* coordinates of the trace centroid (mean *x*, *y*, *z* coordinates); *N* represents the number of traced loci. Chromatin traces with no less than 25 detected loci out of 28 targeted loci were used for radius of gyration calculation for the fine-scale chromatin tracing. Large-scale chromatin tracing data of 40 TADs across the entire human X chromosome were downloaded from a previous work [30]. Chromatin traces with no less than 35 detected loci were used for radius of gyration calculation for the large-scale chromatin tracing.

$$ Radius\ of\ gyration=\sqrt{\frac{\sum \limits_{i=1}^N{\left( xi- xc\right)}^2+{\left( yi- yc\right)}^2+{\left( zi- zc\right)}^2}{N}} $$

## Supplementary Information

**Additional file 1: Supplementary figures**.

**Additional file 2: Supplementary Table 1**. The genomic coordinates of chromatin tracing target regions on human X chromosome.

**Additional file 3: Supplementary Table 2**. Sequences of the chromatin tracing template oligo pools.

**Additional file 4: Supplementary Table 3**. Sequences of the *XIST* RNA FISH template oligo pool.

**Additional file 5: Supplementary Table 4**. Sequences of the intron RNA FISH template oligo pool.

**Additional file 6: Supplementary Table 5**. Sequences of limited-cycle PCR primers, dye-labeled reverse transcription primers and dye-labeled secondary probes.

**Additional file 7:.** Review history.

## Data Availability

The datasets used and/or analyzed during the current study are available from the corresponding author on reasonable request. Previously published data are downloaded from [16, 30, 42, 64, 65]. Codes and analyzed data are available from https://campuspress.yale.edu/wanglab/GenomeBiology2021. Source codes are deposited on Zenodo [66] (10.5281/zenodo.5548146) and Github [67] (https://github.com/SiyuanWangLab/GenomeBiology2021) under the creative commons CC BY-NC license.
