# Supplementary material for: TAD-like single-cell domain structures exist on both active and inactive X chromosomes and persist under epigenetic perturbations
Source: Genome Biol. 2021 Nov 8;22:309. doi: 10.1186/s13059-021-02523-8 (PMC8574027; doi:10.1186/s13059-021-02523-8)
Supplement: Supplementary file 1 — Additional file 1: Supplementary figures. [file 13059_2021_2523_MOESM1_ESM.docx]

**Supplementary Figures**


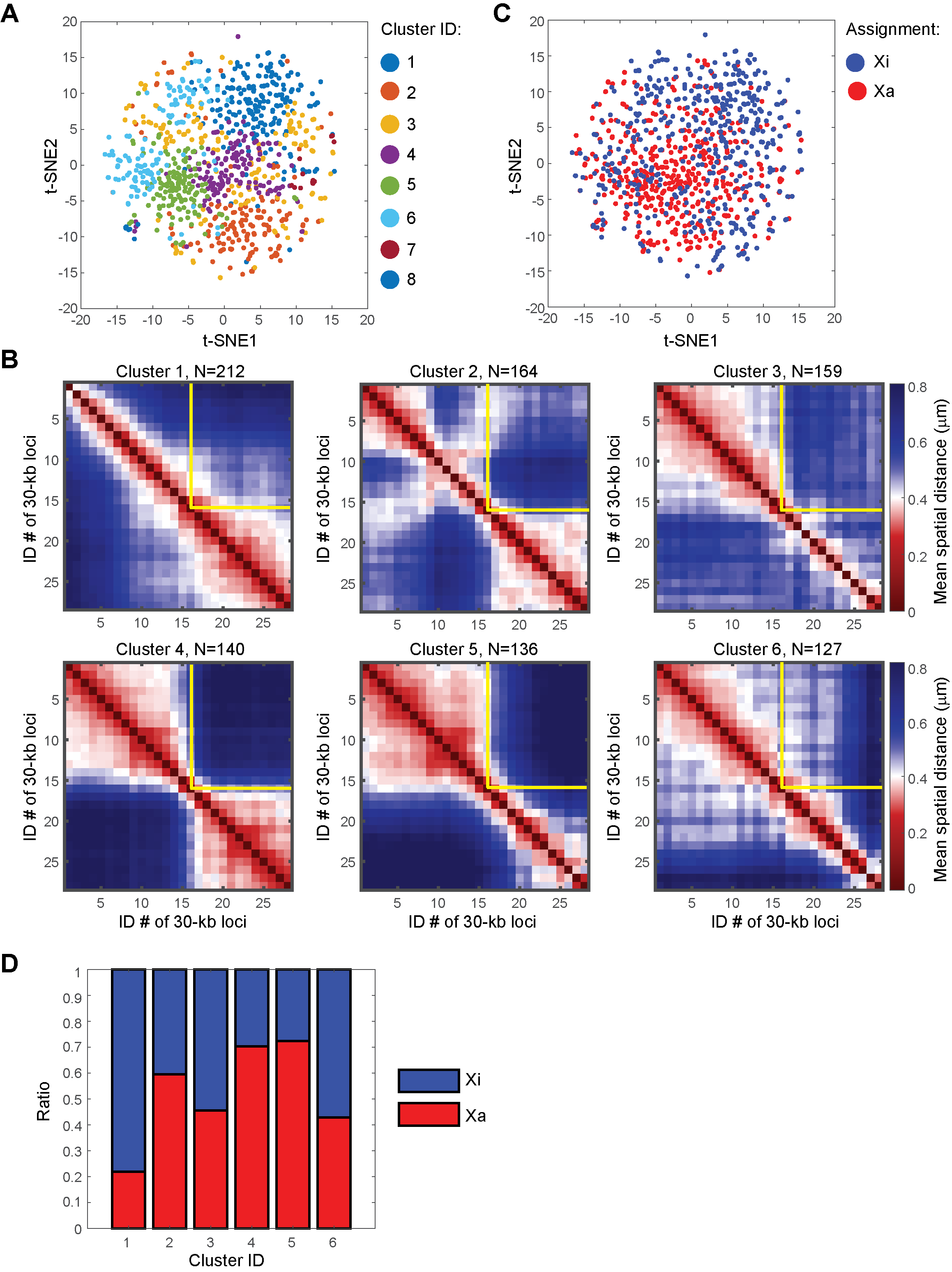


Figure S1. Conformational clustering of chromatin traces. (Target region as stated in Fig. 1.) (A) Louvain-Jaccard clustering result of chromatin traces displayed with t-distributed stochastic neighbor embedding (t-SNE). Each dot represents a chromatin trace. Different pseudo-colors represent different chromatin trace clusters. (B) Mean spatial distance matrices for six major clusters identified in A. The yellow lines represent the ensemble TAD boundary. (C) The same t-SNE plot of chromatin traces as in A but colored by Xi (blue) and Xa (red) identities. (D) Ratio of normalized chromatin trace copy numbers of Xi and Xa in the six major chromatin trace clusters in B. Xi and Xa copy numbers in each cluster were first normalized to the total Xi and Xa copy numbers before calculating the ratio. N = 960 for A, C and D.


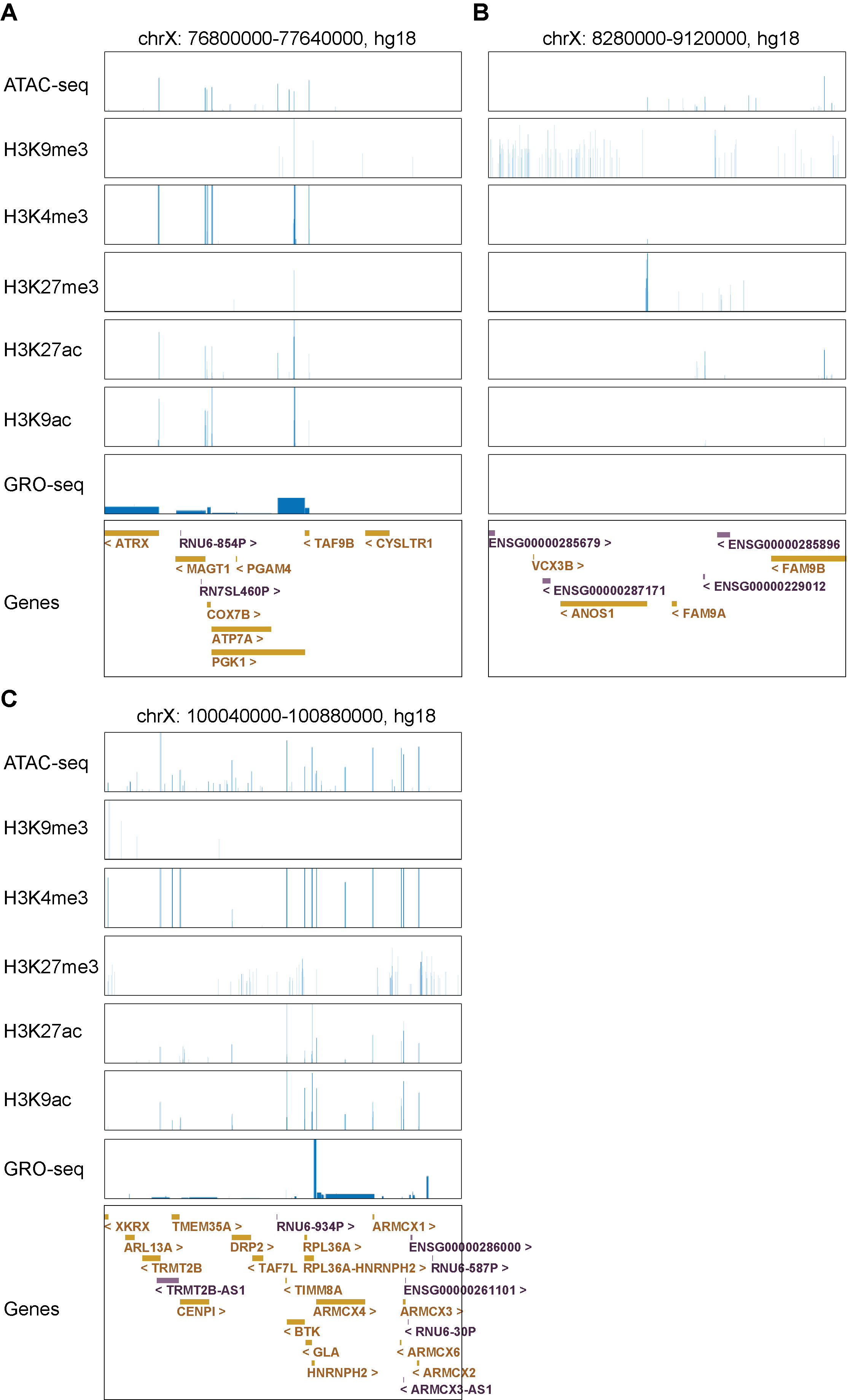


Figure S2. ATAC-seq, ChIP-seq and GRO-seq profiles of the three traced chromatin regions. (A) The main chromatin tracing target region (ChrX: 76,800,000-77,640,000, hg18). (B-C) Two additional representative closed (B, ChrX: 8,280,000-9,120,000, hg18) and open (C, ChrX: 100,040,000-100,880,000, hg18) chromatin tracing regions. ATAC-seq, ChIP-seq and GRO-seq profiles are downloaded from previous work^1–3^.


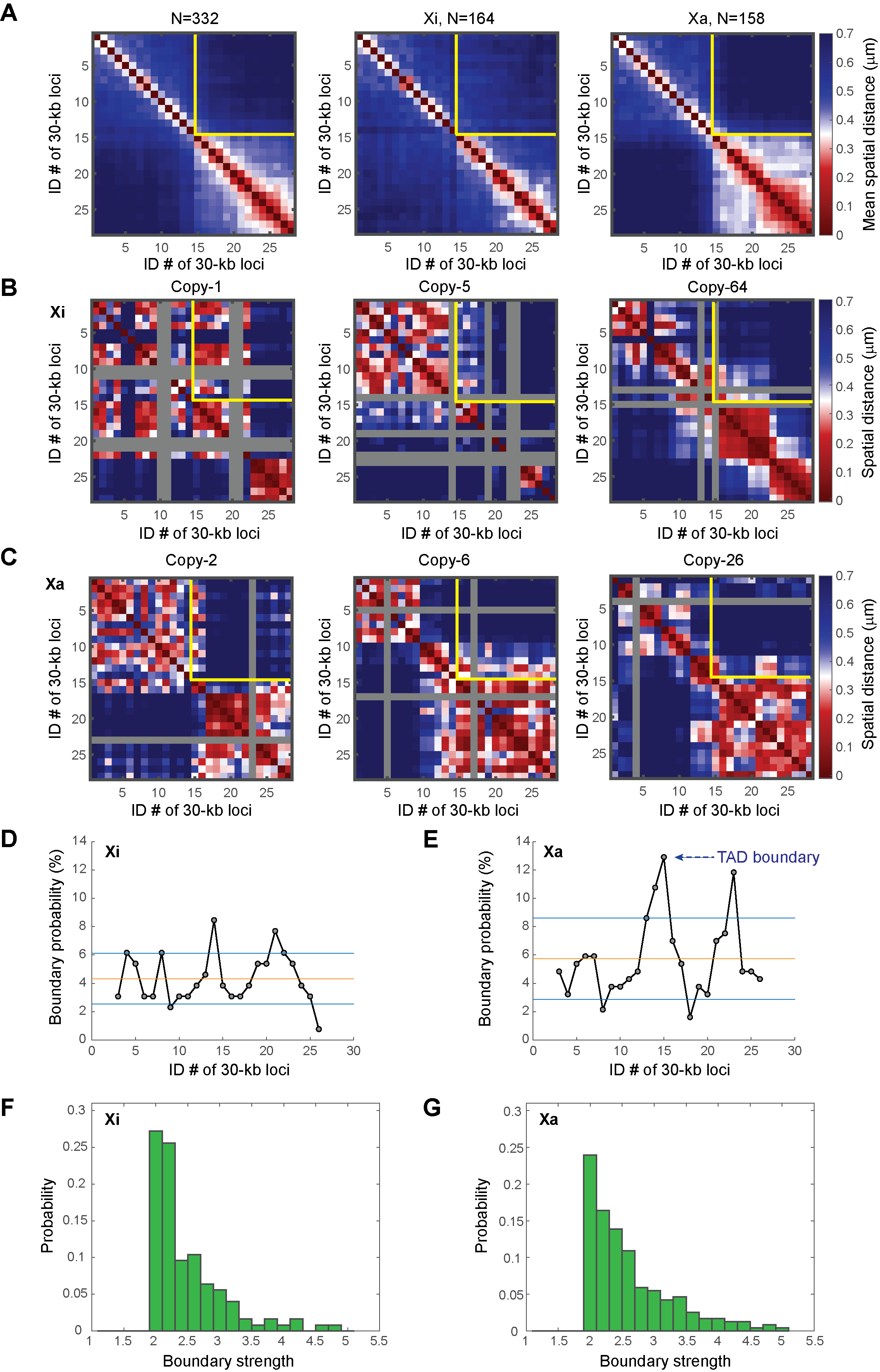


Figure S3. Highly variant TAD-like single-cell domains are present on both inactive and active X chromosomes in a closed chromatin region (ChrX: 8,280,000-9,120,000, hg18) in IMR-90 cells. (A) Mean spatial distance matrices of all chromatin traces (N = 332), Xi traces (N = 164) and Xa traces (N = 158). (B) Examples of individual spatial distance matrices from single copies of inactive X chromosomes. (C) Examples of individual spatial distance matrices from single copies of active X chromosomes. Gray rows and columns in B and C indicate undetected loci. The yellow lines in A-C represent the ensemble TAD boundary. (D) The boundary probabilities of single-cell domains in inactive X chromosomes. (E) The boundary probabilities of single-cell domains in active X chromosomes. The horizontal lines in D and E represent the mean probabilities (orange) and plus/minus one standard deviation (blue). (F) The boundary strengths of single-cell domains in inactive X chromosomes. (G) The boundary strengths of single-cell domains in active X chromosomes.


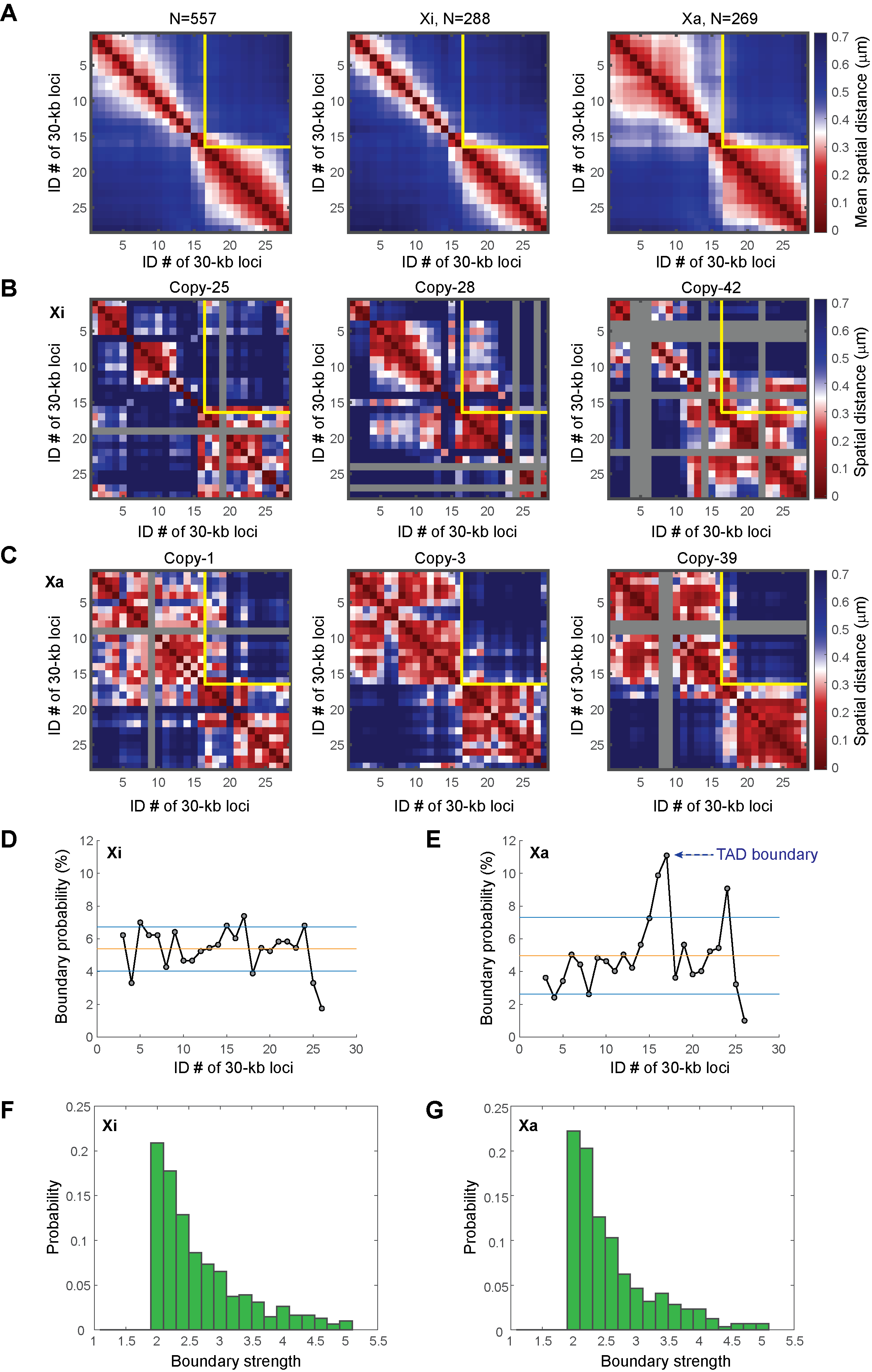


Figure S4. Highly variant TAD-like single-cell domains are present on both inactive and active X chromosomes in an open chromatin region (ChrX: 100,040,000-100,880,000, hg18) in IMR-90 cells. (A) Mean spatial distance matrices of all chromatin traces (N = 557), Xi traces (N = 288) and Xa traces (N = 269). (B) Examples of individual spatial distance matrices from single copies of inactive X chromosomes. (C) Examples of individual spatial distance matrices from single copies of active X chromosomes. Gray rows and columns in B and C indicate undetected loci. The yellow lines in A-C represent the ensemble TAD boundary. (D) The boundary probabilities of single-cell domains in inactive X chromosomes. (E) The boundary probabilities of single-cell domains in active X chromosomes. The horizontal lines in D-E represent the mean probabilities (orange) and plus/minus one standard deviation (blue). (F) The boundary strengths of single-cell domains in inactive X chromosomes. (G) The boundary strengths of single-cell domains in active X chromosomes.


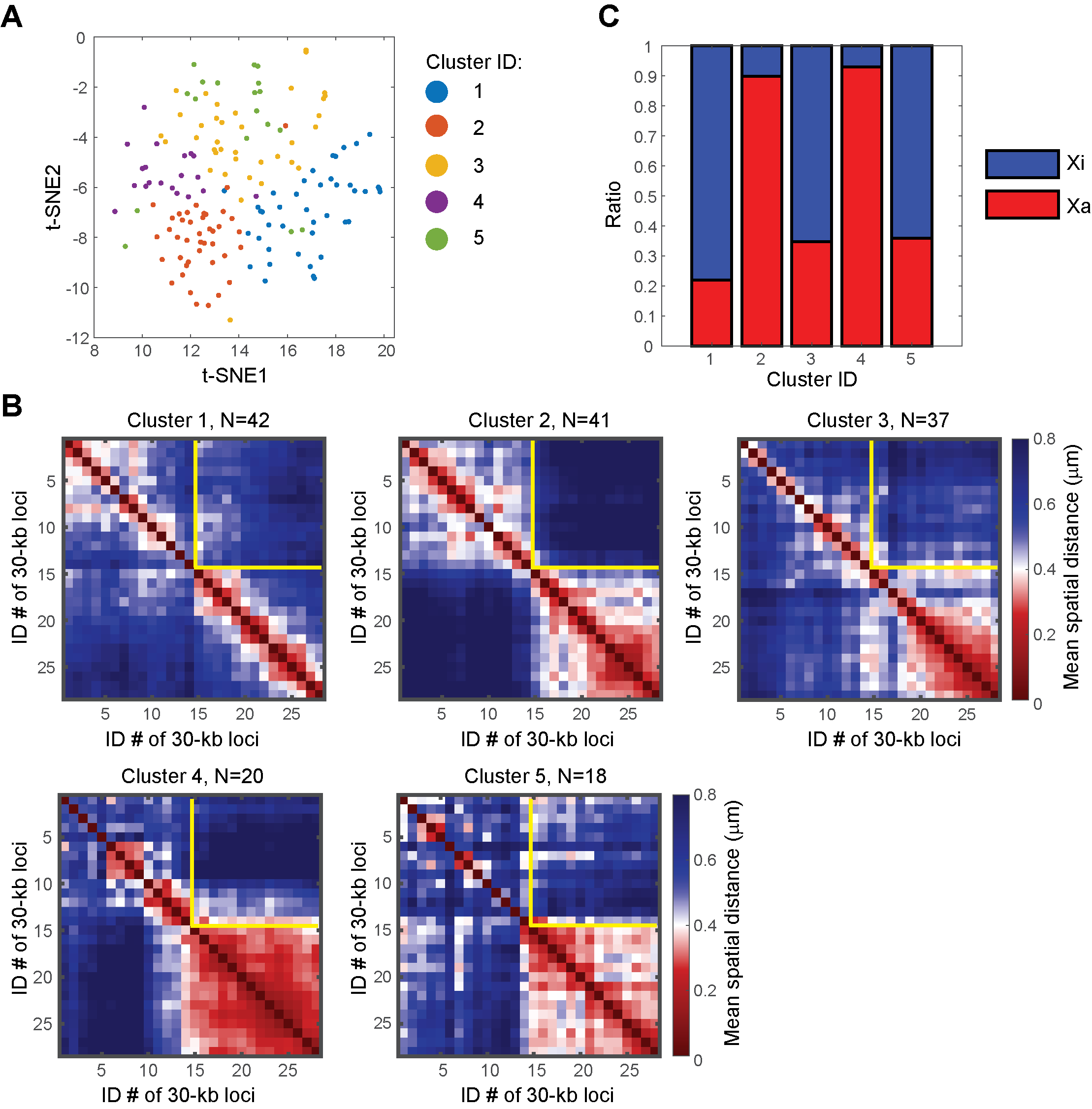


Figure S5. Conformational clustering of chromatin traces of a closed chromatin region (ChrX: 8,280,000-9,120,000, hg18). (A) Louvain-Jaccard clustering result of chromatin traces displayed with t-distributed stochastic neighbor embedding (t-SNE). Each dot represents a chromatin trace. Different pseudo-colors represent different chromatin trace clusters. (B) Mean spatial distance matrices for the five clusters identified in A. The yellow line represents the ensemble TAD boundary. (C) Ratio of normalized chromatin trace copy numbers of Xi and Xa in the five chromatin trace clusters in A. Xi and Xa copy numbers in each cluster were first normalized to the total Xi and Xa copy numbers before calculating the ratio. N = 158 for A and C.


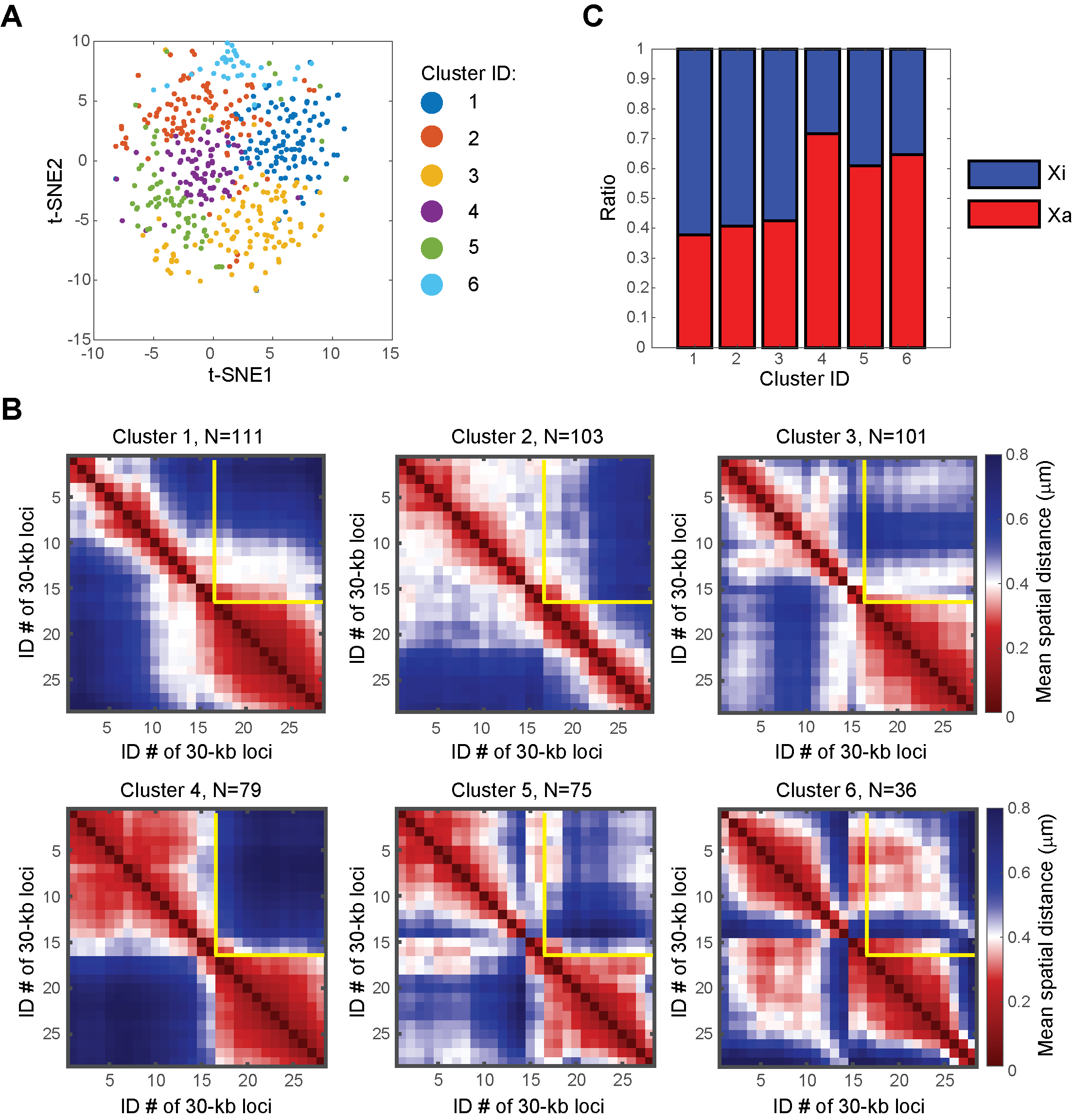


Figure S6. Conformational clustering of chromatin traces of an open chromatin region (ChrX: 100,040,000-100,880,000, hg18). (A) Louvain-Jaccard clustering result of chromatin traces displayed with t-distributed stochastic neighbor embedding (t-SNE). Each dot represents a chromatin trace. Different pseudo-colors represent different chromatin trace clusters. (B) Mean spatial distance matrices for the six clusters identified in A. The yellow line represents the ensemble TAD boundary. (C) Ratio of normalized chromatin trace copy numbers of Xi and Xa in the six chromatin trace clusters in A. Xi and Xa copy numbers in each cluster were first normalized to the total Xi and Xa copy numbers before calculating the ratio. N = 505 for A and C.


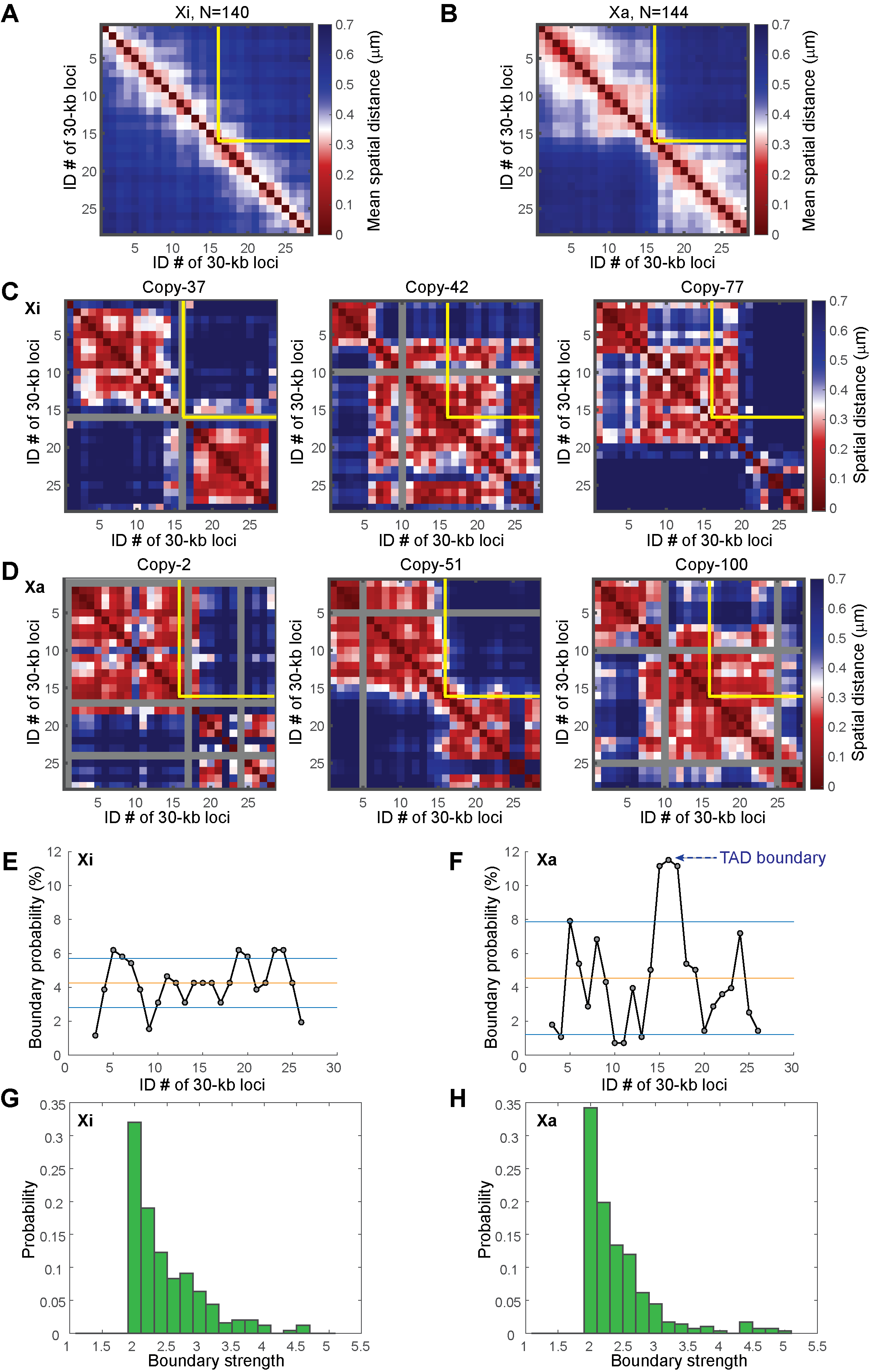


Figure S7. hTERT-RPE-1 cells show similar single-cell domains to those in IMR-90 cells. Data from hTERT-RPE-1 cells are shown in this figure. (Target region as stated in Fig. 1.) (A) Mean spatial distance matrix of inactive X chromosomes. (B) Mean spatial distance matrix of active X chromosomes. (C) Individual spatial distance matrices of single copies of inactive X chromosomes. (D) Individual spatial distance matrices of single copies of active X chromosomes. Gray rows and columns in C and D indicate undetected loci. The yellow lines in A-D represent the ensemble TAD boundary. (E) The boundary probabilities of single-cell domains in inactive X chromosomes. (F) The boundary probabilities of single-cell domains in active X chromosomes. The horizontal lines in E and F represent the mean probabilities (orange) and plus/minus one standard deviation (blue). (G) The boundary strengths of single-cell domains in inactive X chromosomes. (H) The boundary strengths of single-cell domains in active X chromosomes.


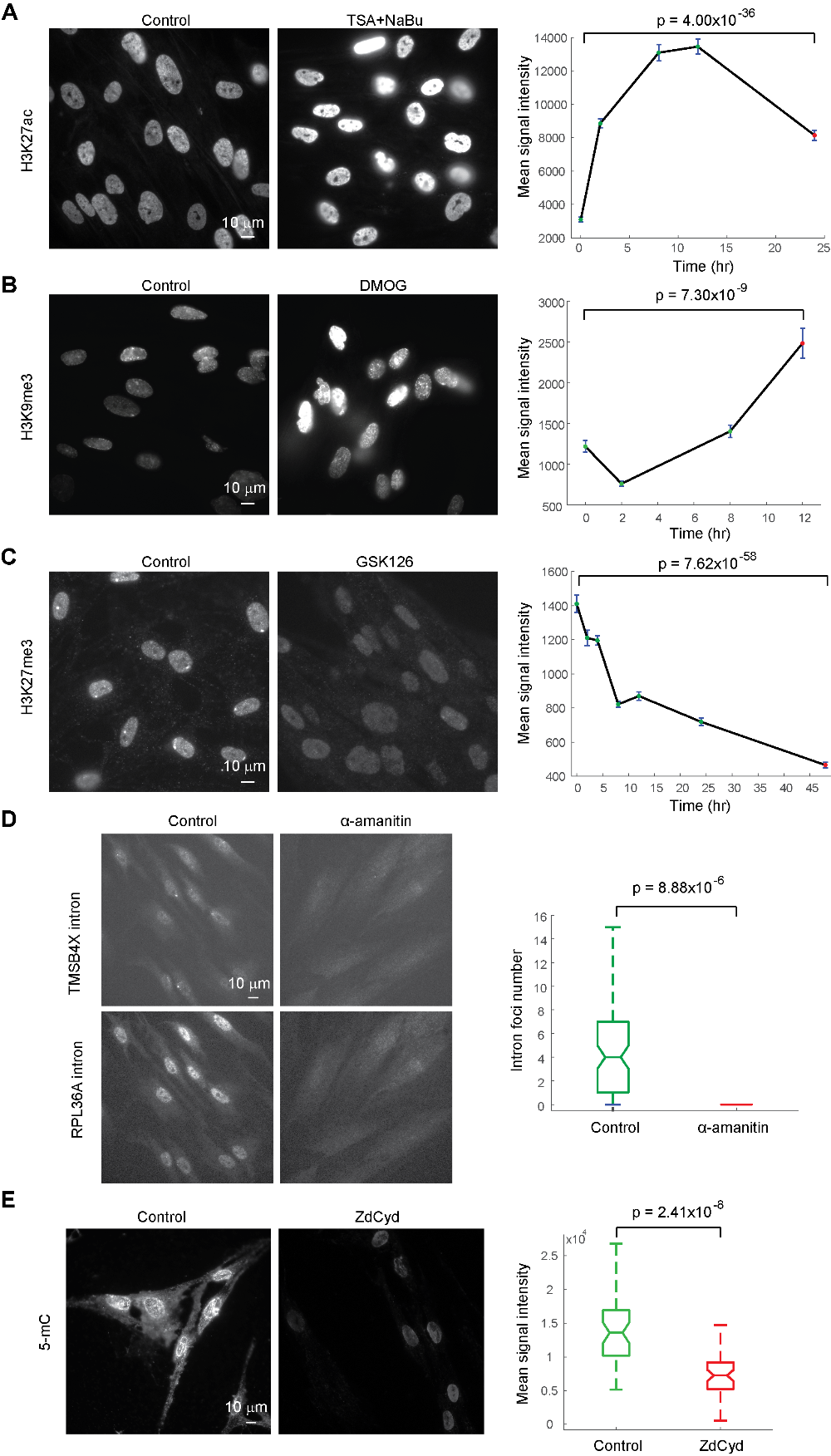


Figure S8. Time course immunofluorescence (IF) staining and intron RNA FISH experiments of different epigenetic perturbations. (A-C) Time course IF measurements of H3K27ac (A), H3K9me3 (B), and H3K27me3 (C) marks after TSA+NaBu (A), DMOG (B) and GSK126 (C) drug treatments. (D) Intron RNA FISH images of X chromosome genes *TMSB4X* and *RPL36A* before and after 8 hours of α-amanitin treatment (left) and quantification of *TMSB4X* intron FISH foci number per cell (right). (E) IF measurements of 5-methyl-cytosine after 24 hours of ZdCyd treatment. The *p* values were calculated using two-sided Student’s t tests. N = 99, 106, 87, 96 and 79 for control, 2 hours, 4 hours, 12 hours and 24 hours of TSA + NaBu in A. N = 66, 44, 59 and 73 for control, 2 hours, 8 hours and 12 hours of DMOG in B. N = 74, 103, 112, 104, 105, 93 and 121 for control, 2 hours, 4 hours, 8 hours, 12 hours, 24 hours and 48 hours of GSK126 in C. N = 94 and 22 for control and α-amanitin in D. N = 49 and 57 for control and 24 hours of ZdCyd in E. Error bars stand for standard error in A-C. Boxes in D and E are defined as in Fig. 3G.


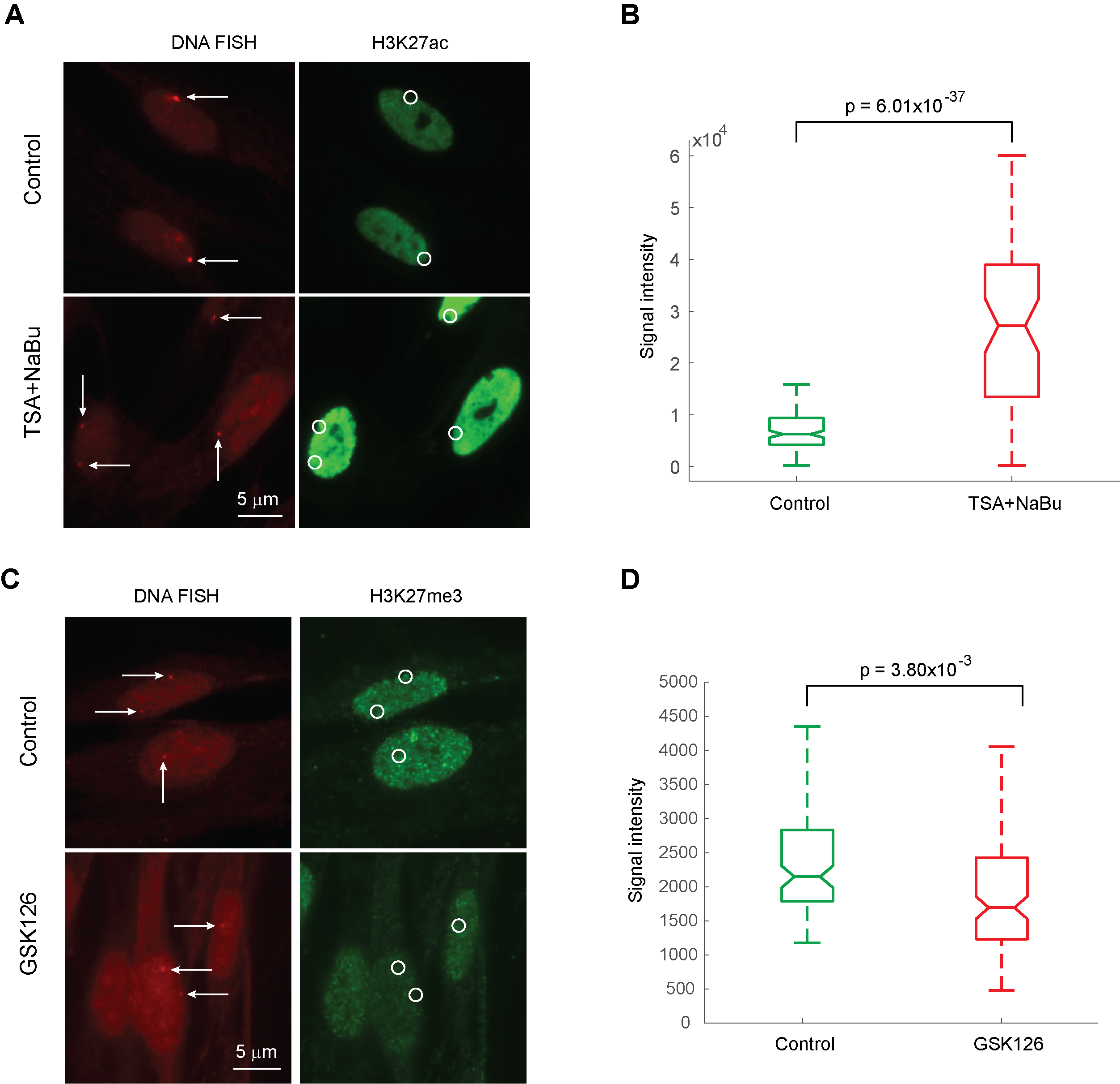


Figure S9. Combined DNA FISH and co-immunofluorescence (IF) staining of different epigenetic perturbations. Treatments with TSA + NaBu (A-B) and GSK126 (C-D) are tested. (A and C) Raw images of DNA FISH and IF staining signals. White arrows indicate DNA FISH foci. White circles indicate the IF signals at the positions of the DNA FISH foci. (B and D) Quantification of IF signal intensity at fitted DNA FISH foci positions. N = 153 and 58 for control and TSA + NaBu treated cells in B. N =104 and 128 for control and GSK126 treated cells in D. The *p* values were calculated using two-sided Student’s t tests. Boxes are defined as in Fig. 3G.


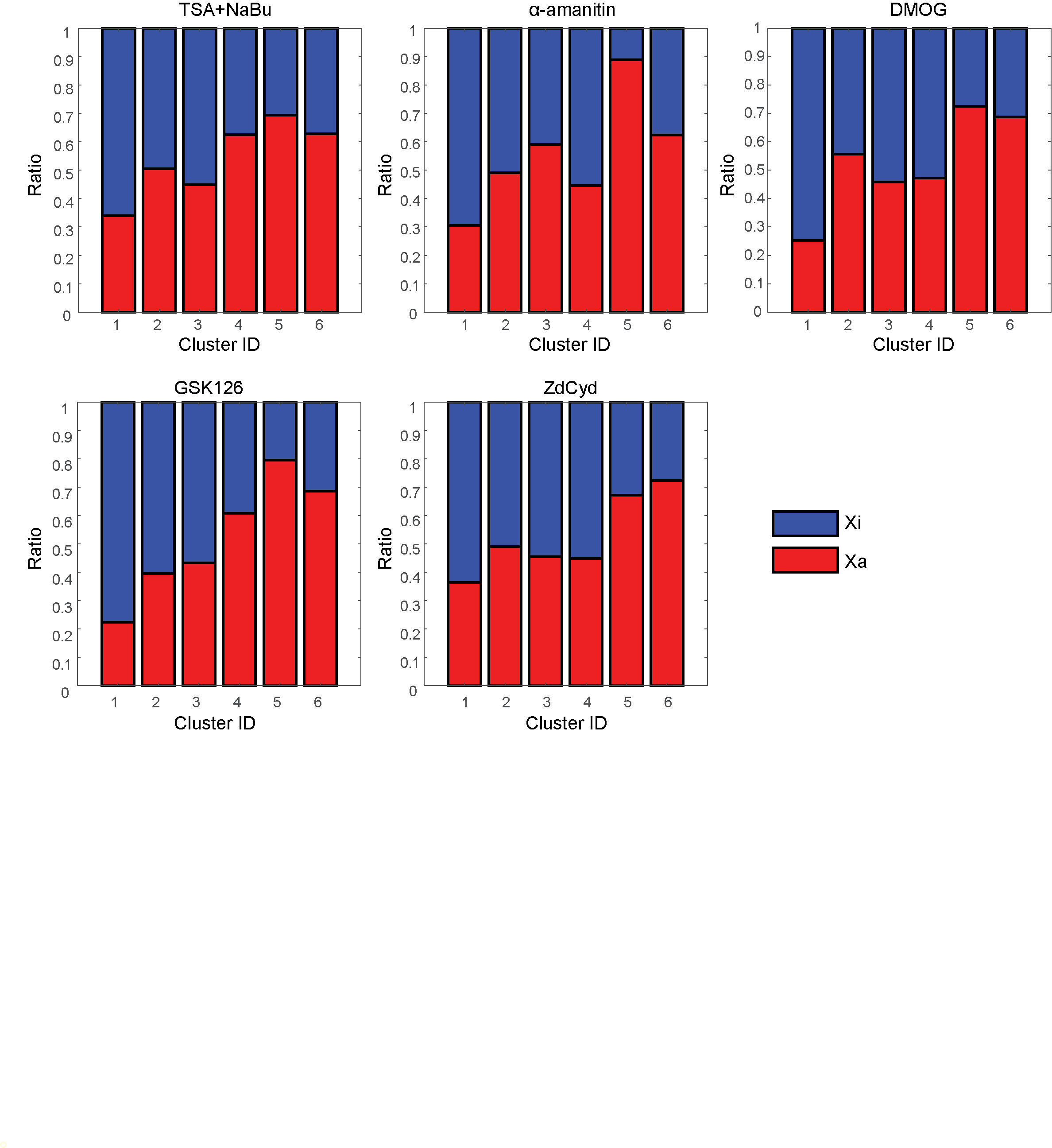


Figure S10. Ratio of normalized chromatin trace copy numbers of Xi and Xa in the six chromatin trace clusters in Figure 4A. Xi and Xa copy numbers in each cluster were first normalized to the total Xi and Xa copy numbers before calculating the ratio.


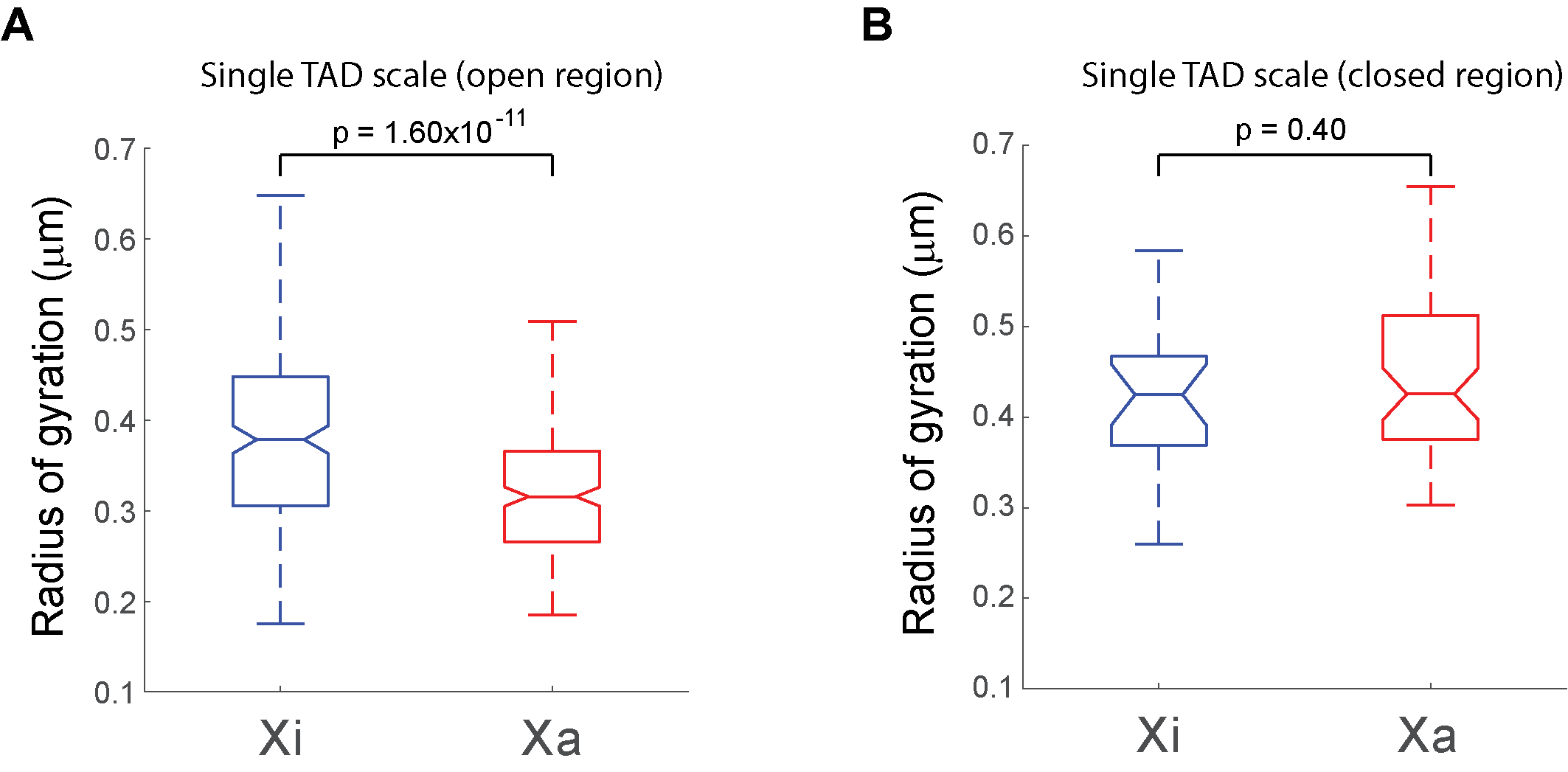


Figure S11. Radii of gyration of Xi and Xa traces in representative open and closed chromatin regions. (A) Radii of gyration of inactive (left, N=216) versus active X chromosomes (right, N=218) from fine-scale chromatin tracing of an 840-kb open chromatin region (ChrX: 100,040,000-100,880,000, hg18). (B) Radii of gyration of inactive (left, N=21) versus active X chromosomes (right, N=57) from an 840-kb closed chromatin region (ChrX: 8,280,000-9,120,000, hg18). The *p* values were calculated using two-sided Student’s t tests. Boxes are defined as in Fig. 3G.

**References**

1. The ENCODE Project Consortium. An integrated encyclopedia of DNA elements in the human genome. *Nature* **489**, 57–74 (2012).

2. Davis, C. A. *et al.* The Encyclopedia of DNA elements (ENCODE): data portal update. *Nucleic Acids Res.* **46**, D794–D801 (2018).

3. Jin, F. *et al.* A high-resolution map of the three-dimensional chromatin interactome in human cells. *Nature* **503**, 290–294 (2013).
