# Supplementary material for: TAD-like single-cell domain structures exist on both active and inactive X chromosomes and persist under epigenetic perturbations
Source: Genome Biol. 2021 Nov 8;22:309. doi: 10.1186/s13059-021-02523-8 (PMC8574027; doi:10.1186/s13059-021-02523-8)
Supplement: Supplementary file 7 — Additional file 7:. Review history. [file 13059_2021_2523_MOESM7_ESM.docx]

Review History

**First round of review**

**Reviewer 1**

**Are you able to assess all statistics in the manuscript, including the appropriateness of statistical tests used?**

Yes, and I have assessed the statistics in my report.

**Comments to author:**

In the manuscript, the authors use chromatin tracing to visualize the chromatin structure of active and inactive X chromosome. Surprisingly, they found the lack of strong ensemble boundary on Xi, and explained by unbiased distribution of boundary positions in single-cell domains, which, however, is somewhat contradict to the canonical bookmark model. In addition, the authors found that perturbations of major epigenetic components did not affect the single-cell domains. The paper is of significance as it deepens our understanding of the formation of TAD-like single-cell domains. The result implies that alternative factors other than loop extrusion and epigenetic landscape may underline the formation of these domains.

Major concerns:

1. The content is too short for an article paper typically in Genome Biology, should be reformatted substantially, and more details shall be added.

2. The author has different treatment time for different drugs, i.e., 8, 24, 48, 12 hours for Amanitin, TSA, GSK and DMOG, respectively. This actually raised a question how to justify the time of treatment? How long the chromatin experienced after the full epigenetic perturbations, as it is known that the chromatin remodeling could take hours. Therefore, a time course data may be more convincing.

3. The conclusion of the Figure 5 remains premature. In Fig5A. the y-axis range from 0.1-.07 with average about 0.3-0.4, while in Fig5B, the average for xi is about 1-1.2. Seemingly suggests that the region(s) examined in Figure 5A may not typical. To me, the 840kb region in Fig5A is more or less locally compacted in both Xi and Xa. An addition analysis may be helpful by comparing the Radius of gyrations of genomic neighbor TADs in Fig5B. If a known open/close region in Xa/Xi region be examined, the pattern should be clearer.

Minor concerns about the paper.

1) Did the authors normalize the distance contact matrix over the expected spatial distances as their previous paper (S. Wang et al., Science, 2016)?

2) For Fig4, are the epigenetic modifications (histone acetylation, histone methylation and transcription machinery) different between Xi and Xa ?

3) In Figure 4E, why N=, 68, 84 for the α-amanitin, while the other Ns > 132, most > 342?

4) The significant have not be indicated in either plots or text, for Figure 4 E and F.

**Reviewer 2**

**Are you able to assess all statistics in the manuscript, including the appropriateness of statistical tests used?**

Yes, and I have assessed the statistics in my report.

**Comments to author:**

This is an interesting study that uses a single-cell resolution multiplexed chromatin tracing technology to study 3D chromatin organization at a selected region on the active and inactive X chromosome of female human cells. Chromatin organization data obtained using this technique recapitulate those seen with Hi-C, for example TAD attenuation is also seen on the Xi relative to Xa. Moreover, the technique used also allows the investigation of chromatin organization at the single-cell level. There the authors find high variability in chromatin organization between different cells, but the aggregated data across all cells analyzed recapitulates TAD attenuation on the Xi. The interesting observation is that despite distinct aggregated patterns, at the single cell level TAD-like single-cell domains are present on both the Xa and Xi. These results were seen in two independent cell lines. To address whether chromatin modifications may be implicated in the formation of the single cell domains, the authors used inhibitors of histone deacetylases and Polycomb, as well as a transcriptional inhibitor. No difference was seen in the formation of single cell domains in the presence of the inhibitors. Based on these results the authors argue that the single-cell domains are independent of variations in "major epigenetic landscapes". Finally, the authors also assess chromatin compaction where no difference was seen between the active and inactive X chromosome for the selected region analyzed. The authors propose that chromatin compaction observed on the Xi comes from a scale above the single-cell domains observed in the individual chromosomes. The claims are mostly novel to the human X chromosomes. The study is of interest to those studying spatial organization of genomic DNA, its relationship with gene regulation and the active or inactive states of the X chromosome. One limitation of the study is the focus on a selected region on the X chromosome and extrapolation of the results and conclusion to the entire X chromosome without further validation of other regions. The results are mostly negative, with no difference in single-cell domains between Xa and Xi or after epi drug inhibition, but those are still valuable to report in my opinion.

I have some questions which upon reading remained unanswered.

1. The work is done at a very selected region that represents a very small proportion of the X chromosome. Can you generalize the findings to the entire X? Can you validate if the findings apply to at least selected other loci? Alternatively, can you acknowledge this in the manuscript in the different sections? You may want to tone down some of the claims. For example: "these results indicate that both Xa and Xi contain single-cell domains of similar features"-> "these results indicate that both Xa and Xi can contain single-cell domains of similar features". Also in the discussion some statements could be modified to acknowledge that what you see at a very specific region and may or may not represents what happens at other regions. This is also relevant for the compaction.

2. Can you replace the term "epigenetic" by "chromatin"? In Many places epigenetic is not defined and it is not clear what is meant and the 'epigenetic' nature of the marks is not certain, therefore it would be better to refer to chromatin instead. Can you replace 'epigenetic profile', 'epigenetic modifications' etc.

3. Bauer et al BioRxiv 2019 (Payer group) have argued from Hi-C data that the Xa contains attenuated TADs. Can you include this study in your manuscript? Can you consider that you may also see attenuated TAD-like structures in some of your analyses?

4. The manuscript is well written.

5. At the start of the results, can you specify how many probes were used?

6. Can you explain what conditional and unconditional probabilities mean? I did not understand this part.

7. Drug treatments - can you specify the length of treatments? The IF validation in Figure S1 are not convincing. First, why is there no H3K27me3 enrichement? Which cell line is this? Not female oterwie we would expect an h3K27me3 Xi-like foci? Can you quantify the signal? Most importantly, does chromatin get hyperacetylated at the region of interest? Same for H3K27me3 and transcription upon EZh2 inhibition and transcription inhibition, respectivelly. Without these controls it is hard to make strong conclusions about the role these mark may play at the region axamined by chromatin tracing. Also alpha amanitin is a transcirptional inhibitor, but it was not validated and also no conclusion was made on the role of transcription in single-cell domains. You could expand there.

8. Radii of gyration. Can you explain this better? I did not understand it.

Additional comments:

9. Xist should be changed to its human nomenclature: XIST.

10. Page 4, line 26. Specify what cells are these "human female IMR-90 cell line" here (lung fibroblasts, mentioned below).

11. Figure 1C and 1D. Reference the study from which IMR-90 Hi-C data was reanalyzed in the panels. For example, write between brackets the published reference next to "Hi-C number of reads".

12. Figure 2C and 2D. Switch 2C to 2D and 2D to 2C. Or, switch explanation in text.

13. Page 6, line 21-22. Explain Xa first and Xa later (following the order of the figures).

14. Figure 3. This figure should be merged with figure 2 (or shown as supplemental figure) since it does not make a new point but rather extends figure 2 findings to another human cell line.

15. Figure S1. This figure should show the immunofluorescence images in the order that appear on the text TSA + NaBu, DMOG and GSK126.

16. Figure 4A-D. Add the treatment applied to each panel in the figure. In addition, add examples for both X chromosome per condition.

17. Figure 4 E-F. DMOG seems to increase boundary frequency and strength, could you check if this increase is non-significant or significant? Also, run statistical tests for all conditions.

18. Figure 4 E-F. GSK126 treated cells do not have allelic resolution information. Why? Can you please add allelic resolution to this condition?

19. Page 6. Perturbation experiment, similar to point above. Add information about which cell line and how long these inhibitors were applied.

20. Page 6, line 34-35. Clearly indicate that both (TSA and NaBu) inhibitors are added together.

21. Page 6. Perturbation experiment. Can you include a DNA methylation perturbation experiment to ask if DNA methylation can affect single-cell domains?

22. Figure 5. Indicate in panel A and B from where these radius of gyration come from (single TADs versus TAD-to-chromosome). Fig. 5B. Add between brackets on the y-axis the study from which this data was re-analysed (reference 30).

23. Discussion: Can you hypothesized which molecular mechanism could affect single-cell domains in the discussion?

24. Discussion: Discuss your results in light of these recent pre-prints: 1) Bauer/Payer 2020 bioRxiv (TAD-like compartmentalization persists on the Xi) and 2) Takei/Cai 2021 bioRxiv (single-cell chromosome domain structures on Xa and Xi chromosomes) both studies performed in mouse.

**Reviewer 3**

**Are you able to assess all statistics in the manuscript, including the appropriateness of statistical tests used?**

There are no statistics in the manuscript.

**Comments to author:**

The authors used chromatin tracing to analyze the genome organization in human female cells. They compared the active and inactive X chromosomes. They found TAD-like single cell domains in both Xa and Xi cells despite the drastic difference between the Xi and Xa chromosomes at the bulk level: TADs are observed in Xa but not in Xi chromosome. These single cell domains are highly variable across cells. The authors repeated these experiments in two different cell types (IMR90 and hTERT-RPE-1 cells) to confirm that this observation is not specific to one particular cell type. Furthermore, they applied several inhibitors to perturb histone modifications and transcription. They found that the single cell domains are resistant to such perturbations. They also showed that the Xi is more compact than Xa at the large but not fine scale. This study reports an interesting and exciting observation. This manuscript can be further improved by addressing the following issues.

1. As the single cell domains exist for both Xa and Xi, more discussion is needed to explain why Xa shows TADs but Xi not at the bulk level. There is one sentence in the paper to state that the Xi single domain boundaries are more uniformly distributed while the Xa boundaries occur at the TAD boundaries. This statement requires a quantitative measurement to support it.

2. Related to the above point, do cells fall into different clusters with similar domain patterns? The variation analysis of the single cell domains can be done at the cluster level if there are distinct clusters. Showing bulk TAD boundaries would also be helpful to illustrate the variation of the single cell domains.

3. Are CTCF and cohesin enriched at the single cell domain boundaries?

4. The interpretation of the epigenetic inhibitor results requires some caution. These inhibitors affect the global histone methylation or acetylation. But to draw a concrete conclusion that epigenetic modification or transcription does not affect single cell domains requires mapping histone modifications in single cells for Xi and Xa after inhibitor treatment. It would also be interesting to cluster the single cell domains after perturbation and compare them with the untreated cells.

5. The imaging data should be available for download.

Minor:

1. reference genome in Methods and Table S1 is hg18. Is it hg38?

**Authors Response**

**Point-by-point responses to the reviewers’ comments:**

Reviewer #1:

In the manuscript, the authors use chromatin tracing to visualize the chromatin structure of active and inactive X chromosome. Surprisingly, they found the lack of strong ensemble boundary on Xi, and explained by unbiased distribution of boundary positions in single-cell domains, which, however, is somewhat contradict to the canonical bookmark model. In addition, the authors found that perturbations of major epigenetic components did not affect the singlecell domains. The paper is of significance as it deepens our understanding of the formation of TAD-like single-cell domains. The result implies that alternative factors other than loop extrusion and epigenetic landscape may underline the formation of these domains.

*Reply: We thank the reviewer for the insightful and comprehensive evaluations of our work! Below are the point-by-point responses to the reviewer’s concerns.*

Major concerns: 1. The content is too short for an article paper typically in Genome Biology, should be reformatted substantially, and more details shall be added.

*Reply: We thank the reviewer for the suggestion. In the revised manuscript, we added substantially more experiments, analyses and method details to better justify the article paper format, and reformatted the manuscript according to the Genome Biology journal instructions.*

2. The author has different treatment time for different drugs, i.e., 8, 24, 48, 12 hours for Amanitin, TSA, GSK and DMOG, respectively. This actually raised a question how to justify the time of treatment? How long the chromatin experienced after the full epigenetic perturbations, as it is known that the chromatin remodeling could take hours. Therefore, a time course data may be more convincing.

*Reply: We thank the reviewer for the great suggestion. To justify the time of drug treatment, we performed time course immunofluorescence (IF) staining experiments of H3K27me3, H3K27ac, and H3K9me3 marks after GSK126, TSA+NaBu and DMOG drug treatments, and performed intron RNA FISH experiments to measure transcription strength after α-amanitin treatment (Response Figure 1). The measurements showed that H3K27ac IF intensity significantly increased after 2-24 hours of TSA+NaBu (histone deacetylase inhibitor) treatment (Response Figure 1A). H3K9me3 IF intensity significantly increased after 12 hours of DMOG (HIF-prolylhydroxylase inhibitor) treatment (Response Figure 1B). H3K27me3 IF intensity significantly decreased after 48 hours of GSK126 (EZH2 methyltransferase inhibitor) treatment (Response Figure 1C). Intron RNA FISH signals (nascent transcripts) of X chromosome genes TMSB4X and RPL36A significantly decreased after 8 hours of α-amanitin treatment (Response Figure 1D). To conclude, we showed that the treatment time in the manuscript (8, 24, 48, 12 hours for α-amanitin, TSA+NaBu, GSK126 and DMOG respectively) is sufficient for the transcription or epigenetic state perturbations. We newly added these results in a new Figure S6 of the manuscript.*

***Response Figure 1. Time course immunofluorescence (IF) staining and intron RNA FISH experiments of different epigenetic perturbations.*** *(****A-C****) Time course IF staining experiments of H3K27ac, H3K9me3, and H3K27me3 marks after TSA+NaBu (****A****), DMOG (****B****) and GSK126 (****C****) drug treatments. (****D****) Intron RNA FISH images of X chromosome genes TMSB4X and RPL36A before and after 8 hours of α-amanitin treatment (left) and quantification of TMSB4X intron FISH foci number per cell (right). The p values were calculated using two-sided Student’s t tests.* *N = 99, 106, 87, 96 and 79 for control, 2 hours, 4 hours, 12 hours and 24 hours of TSA + NaBu in A. N = 66, 44, 59 and 73 for control, 2 hours, 8 hours and 12 hours of DMOG in B. N = 74, 103, 112, 104, 105, 93 and 121 for control, 2 hours, 4 hours, 8 hours, 12 hours, 24 hours and 48 hours of GSK126 in C. N = 94 and 22 for control and α-amanitin in D. Error bars in A-C stand for standard error. Boxes in D are defined as in Fig. 3G.*

3. The conclusion of the Figure 5 remains premature. In Fig5A. the y-axis range from 0.1-.07 with average about 0.3-0.4, while in Fig5B, the average for xi is about 1-1.2. Seemingly suggests that the region(s) examined in Figure 5A may not typical. To me, the 840kb region in Fig5A is more or less locally compacted in both Xi and Xa. An addition analysis may be helpful by comparing the Radius of gyrations of genomic neighbor TADs in Fig5B. If a known open/close region in Xa/Xi region be examined, the pattern should be clearer.

*Reply: We thank the reviewer for the constructive suggestion. We agree that additional analyses of neighboring TADs will be more convincing. Therefore, we designed new chromatin tracing probes targeting 28 consecutive 30-kb regions along two additional 840-kb regions across TAD boundaries on the X chromosome. The two new regions are representative “closed” and “open” chromatin regions respectively (Response Figure 2B-C, new Figure S2) 1 . We performed the same chromatin tracing procedure in both regions and analyzed their radii of gyration in the Xi and Xa chromosomes. The results showed that the radii of gyration were similar between the Xi and Xa chromatin for the two additional 840-kb regions, and had ranges similar to the that of the previous 840-kb region (Response Figure 3, new Figure S8), consistent with our previous measurements and conclusion. We note the previous Figure 5B displays the radii of gyration of the entire Xa and Xi chromosomes, thus the difference between the ranges of Figure 5A and Figure 5B are likely due to the different targeted genomic lengths (840kb versus whole X chromosome). Interestingly, in the new 840kb open region, although the fold difference is small, the Xa chromatin appears to be slightly more compact than the Xi chromatin (Response Figure 2, p =1.60x10-11). This more compact Xa chromatin configuration may be due to cis-regulatory interactions for transcription at the fine scale.*

*In summary, these additional analyses support the conclusion in the manuscript that chromatin compaction associated with X inactivation occur at a length scale above that of TADs and single-cell domains.*

***Response Figure 2.*** ***ATAC-seq, ChIP-seq and GRO-seq profiles of the three traced chromatin regions.*** *(A) The main chromatin tracing target region (ChrX: 76,800,000-77,640,000, hg18). (B-C) Two additional representative closed (B, ChrX: 8,280,000-9,120,000, hg18) and open (C, ChrX: 99,080,000-99,920,000, hg18) chromatin tracing regions. ATAC-seq, ChIP-seq and GRO-seq* *profiles are downloaded from previous works^1–3^.*

***Response Figure 3. Radii of gyration of Xi and Xa traces in representative open and closed chromatin regions.*** *(A) Radii of gyration of inactive (left, N=216) versus active X chromosomes (right, N=218) from fine-scale chromatin tracing of an 840-kb open chromatin region (ChrX: 99,080,000-99,920,000, hg18). (B) Radii of gyration of inactive (left, N=21) versus active X chromosomes (right, N=57) from an 840-kb closed chromatin region (ChrX: 8,280,000-9,120,000, hg18).* *The p values were calculated using two-sided Student’s t tests. Boxes are defined as in Fig. 3G.*

Minor concerns about the paper.

1) Did the authors normalize the distance contact matrix over the expected spatial distances as their previous paper (S. Wang et al., Science, 2016)?

*Reply: We thank the reviewer for pointing this out. The mean spatial distance matrices in Figure 1 were showing average spatial distances and were not normalized over the expected spatial distances as in our previous paper analyzing A-B compartmentalization (S. Wang et al., Science, 2016). We newly normalized the mean spatial distances over the expected spatial distances derived from the power-law fitting of spatial versus genomic distances, and displayed the normalized spatial distance matrix in Response Figure 4. As in the raw mean spatial distance matrices, at the ensemble level, the Xa normalized matrix revealed strong long-range interactions and the TAD boundary, while the Xi matrix showed attenuated long-range interactions and TAD boundary (Response Figure 4). To be consistent with a previous paper first reporting the TAD-like single-cell domains4 , we opt to use the raw distance matrices in the manuscript.*

***Response Figure 4. Normalized spatial distance matrices of IMR-90 cell lines using chromatin traces of the first chromatin tracing target region (ChrX: 76,800,000-77,640,000, hg18).*** *The normalized matrices of all chromatin traces (left, N =1038), Xa traces (middle, N = 519), and Xi traces (right, N = 519) were derived by normalizing the mean spatial distance matrices to the expected spatial distances calculated from a spatial-versus-genomic distance power-law fitting as established in (S. Wang et al., Science, 2016).*

2) For Fig4, are the epigenetic modifications (histone acetylation, histone methylation and transcription machinery) different between Xi and Xa?

*Reply: We thank the reviewer for the question. Although there are previous reports (as reviewed in Ref.5) that active epigenetic marks and transcription are depleted in Xi in comparison to Xa, and repressive epigenetic marks are enriched in Xi in comparison to Xa, to strictly answer how the epigenetic modifications vary between Xi and Xa in the main IMR-90 cell line used in this study, we searched for published Xa and Xi-specific allelic ChIP-seq and allelic RNA-seq data for IMR-90. Unfortunately, we were not able to find the corresponding reports. Given the limited revision time and resources in the lab, we would like to ask for the reviewer’s permission for us to perform these measurements in a future study.*

3) In Figure 4E, why N=, 68, 84 for the α-amanitin, while the other Ns > 132, most > 342?

*Reply: We thank the reviewer for pointing this out. To get a higher N number for the αamanitin treatment condition, we collected more datasets and updated Figure 4E (now Figure 3F in the revised manuscript). We now have an N number of 163 for Xi and 188 for Xa in the αamanitin treatment condition.*

4) The significant have not be indicated in either plots or text, for Figure 4 E and F.

*Reply: We thank the reviewer for pointing this out. We now add the p-values in figure legends of Figure 4E-4F (now Figure 3F-3G in the revised manuscript): “(F) Average boundary frequencies with or without TSA + NaBu, DMOG, GSK126, α-amanitin and ZdCyd treatments. Error bars stand for standard deviation. For the Xi and Xa bars in the treated groups, from left to right, p = 0.70, 0.50, 1.55x10^-5^, 8.30x10^-8^, 0.14, 0.19, 0.17, 0.51, 0.17, 0.95, in comparison to control. (G) Boundary strengths with or without TSA + NaBu, DMOG, GSK126, α-amanitin and ZdCyd treatments. For the Xi and Xa boxes in the treated groups, from left to right, p = 0.41, 0.034, 6.80x10^-3^, 1.14x10^-9^, 0.39, 0.20, 0.41, 0.77, 0.50, 0.77, in comparison to control. For each box, the horizontal lines from top to bottom represent the nonoutlier maximum, 75% quantile, median, 25% quantile and non-outlier minimum. Outliers are defined as values that are more than 1.5 times the interquartile range away from the bottom or top of the box. In F and G from left to right: N = 483, 477, 182, 201, 248, 319, 186, 183, 163, 188, 111, 115. All p values were calculated using two-sided Student’s t tests.”*

Reviewer #2:

This is an interesting study that uses a single-cell resolution multiplexed chromatin tracing technology to study 3D chromatin organization at a selected region on the active and inactive X chromosome of female human cells. Chromatin organization data obtained using this technique recapitulate those seen with Hi-C, for example TAD attenuation is also seen on the Xi relative to Xa. Moreover, the technique used also allows the investigation of chromatin organization at the single-cell level. There the authors find high variability in chromatin organization between different cells, but the aggregated data across all cells analyzed recapitulates TAD attenuation on the Xi. The interesting observation is that despite distinct aggregated patterns, at the single cell level TAD-like single-cell domains are present on both the Xa and Xi. These results were seen in two independent cell lines. To address whether chromatin modifications may be implicated in the formation of the single cell domains, the authors used inhibitors of histone deacetylases and Polycomb, as well as a transcriptional inhibitor. No difference was seen in the formation of single cell domains in the presence of the inhibitors. Based on these results the authors argue that the single-cell domains are independent of variations in "major epigenetic landscapes". Finally, the authors also assess chromatin compaction where no difference was seen between the active and inactive X chromosome for the selected region analyzed. The authors propose that chromatin compaction observed on the Xi comes from a scale above the single-cell domains observed in the individual chromosomes. The claims are mostly novel to the human X chromosomes. The study is of interest to those studying spatial organization of genomic DNA, its relationship with gene regulation and the active or inactive states of the X chromosome. One limitation of the study is the focus on a selected region on the X chromosome and extrapolation of the results and conclusion to the entire X chromosome without further validation of other regions. The results are mostly negative, with no difference in single-cell domains between Xa and Xi or after epi drug inhibition, but those are still valuable to report in my opinion.

*Reply: We thank the reviewer for the very constructive and supportive comments of our work! We now provide the point-to-point responses to the reviewer’s questions.*

I have some questions which upon reading remained unanswered. 1. The work is done at a very selected region that represents a very small proportion of the X chromosome. Can you generalize the findings to the entire X? Can you validate if the findings apply to at least selected other loci? Alternatively, can you acknowledge this in the manuscript in the different sections? You may want to tone down some of the claims. For example: "these results indicate that both Xa and Xi contain single-cell domains of similar features"-> "these results indicate that both Xa and Xi can contain single-cell domains of similar features". Also in the discussion some statements could be modified to acknowledge that what you see at a very specific region and may or may not represents what happens at other regions. This is also relevant for the compaction.

*Reply: We thank the reviewer for the very constructive suggestion. To test whether our observations can be generalized to at least selected other loci, we analyzed chromatin conformations of two additional 840-kb regions across TAD boundaries on the X chromosome (Response Figures 5-6, new Figure S3-4) and included the new findings in the manuscript as highlighted below (Page 8):*

*To study whether our findings can be generalized to other regions on the human X chromosome, we profiled chromatin organizations of two additional 840-kb regions spanning TAD boundaries identified from Hi-C^6,7^. One additional target region corresponded to a closed chromatin region with low signals in assay for transposase-accessible chromatin using sequencing (ATAC-seq) and high H3K9me3 ChIP-seq signals, whereas the other target region was an open chromatin region with high ATAC-seq signals and active gene transcription (Fig. S2). To compare chromatin conformations of Xi and Xa at the ensemble level, we generated separate mean spatial distance matrices for Xi and Xa. Similar to the target region profiled above (Fig. 1E), the Xi matrices showed attenuated TAD boundaries while the Xa matrices showed prominent TAD boundaries in both the closed and open regions (Fig. S3A, S4A). To compare potential singlecell domains in individual copies of Xi and Xa, we generated individual spatial distance matrices of single copies of Xi and Xa. Both individual copies of Xi and Xa traces showed single-cell domains with sharp boundaries in both the closed and open regions (Fig. S3B-C, S4B-C). Single-cell domain boundary positions were highly variant in both Xi and Xa traces (Fig. S3B-C, S4B-C). Quantifications of boundary probabilities suggested that single-cell domain boundaries in Xi were significantly more uniformly distributed along the traced regions than those in Xa (p = 0.026 for the closed region and p = 0.0098 for the open region, F-tests), whereas single-cell domain boundaries in Xa preferentially resided at the ensemble TAD boundaries (Fig. S3D-E, S4D-E). Moreover, quantifications of boundary strengths indicated that Xi and Xa single-cell domains had similar boundary strengths (Fig. S3F-G, S4F-G). These analyses revealed that the organizational features of ensemble Xi and Xa chromatin and the existence of single-cell domains are not limited to one X chromosome region.*

*Because our analyses cover only selected regions but not the entire X chromosome, we further toned down our conclusion by using “can” and added the following sentences in the discussion session as suggested by the reviewer (Page 12):*

*“Whether our observations can be generalized to the entire X chromosome or the whole genome is also unknown.”*

***Response Figure 5. Highly variant TAD-like single-cell domains are present on both inactive and active X chromosomes in a closed chromatin region (ChrX: 8,280,000-9,120,000, hg18) in IMR-90 cells.*** *(A) Mean spatial distance matrices of all chromatin traces (N = 332), Xi traces (N = 164) and Xa traces (N = 158). (B) Examples of individual spatial distance matrices from single copies of inactive X chromosomes. (C) Examples of individual spatial distance matrices from single copies of active X chromosomes. Gray rows and columns in A and B indicate undetected loci. The yellow lines in A-C represent the ensemble TAD boundary. (D) The boundary probabilities of single-cell domains in inactive X chromosomes. (E) The boundary probabilities of single-cell domains in active X chromosomes.* *The horizontal lines in D and E represent the mean probabilities (orange) and plus/minus one standard deviation (blue). (F) The boundary strengths of single-cell domains in inactive X chromosomes. (G) The boundary strengths of single-cell domains in active X chromosomes.*

***Response Figure 6. Highly variant TAD-like single-cell domains are present on both inactive and active X chromosomes in an open chromatin region (ChrX: 99,080,000-99,920,000, hg18) in IMR-90 cells.*** *(A) Mean spatial distance matrices of all chromatin traces (N = 557), Xi traces (N = 288) and Xa traces (N = 269). (B) Examples of individual spatial distance matrices from single copies of inactive X chromosomes. (C) Examples of individual spatial distance matrices from single copies of active X chromosomes. Gray rows and columns in A and B indicate undetected loci. The yellow lines in A-C represent the ensemble TAD boundary. (D) The boundary probabilities of single-cell domains in inactive X chromosomes. (E) The boundary probabilities of single-cell domains in active X chromosomes. The horizontal lines in D and E represent the mean probabilities (orange) and plus/minus one standard deviation (blue). (F) The boundary strengths of single-cell domains in inactive X chromosomes. (G) The boundary strengths of single-cell domains in active X chromosomes.*

2. Can you replace the term "epigenetic" by "chromatin"? In Many places epigenetic is not defined and it is not clear what is meant and the 'epigenetic' nature of the marks is not certain, therefore it would be better to refer to chromatin instead. Can you replace 'epigenetic profile', 'epigenetic modifications' etc.

*Reply: We thank the reviewer for pointing this out. Indeed after initial submission we also increasingly felt that the undefined terms including “epigenetic interaction landscape”, “epigenetic landscape”, and “epigenetic profile” may be ambiguous to the readers. While we used these terms to describe profiles of generalized epigenetic features such as the distribution of epigenetic reader proteins and chromatin interaction instances mediated by the proteins in individual copies of chromatin, some readers may be used to a much narrower definition of these terms limited to epigenetic marks on histones or DNA. In this version, we removed/revised these terms as the reviewer suggested to avoid readers confusing these terms with a more narrowly defined epigenetic mark profile. We also added more descriptions and discussions of the previous computational model and clarified the relationship between our new terms and the terms and components used in the model.*

3. Bauer et al BioRxiv 2019 (Payer group) have argued from Hi-C data that the Xa contains attenuated TADs. Can you include this study in your manuscript? Can you consider that you may also see attenuated TAD-like structures in some of your analyses?

*Reply: We thank the reviewer for the suggestion. As the reviewer pointed out, the Bauer et al biorxiv 2019 study (now published in Nature Communications^8^) showed from bulk Hi-C data that TADs on Xi appeared attenuated but not lost in mouse. To test if Xi contains attenuated TADs in our study, we checked the mean spatial distance matrices of Xi in all three of our target regions. At the population-averaged level, the target TADs on Xi appeared attenuated, but TAD features are not completely lost (Figure 1E, S3A, S4A, S5A), which was consistent with the bulk Hi-C studies in mouse ^8–10^. As the reviewer suggested, we newly added this study in the citations of the revised manuscript, and pointed out that there are attenuated TAD structures on Xi (Page 6): “Next, to compare the chromatin folding conformations between Xi and Xa, we generated separate mean spatial distance matrices for Xi and Xa. The Xi matrix showed an attenuation of the TAD boundary, but some residual TAD features still persisted, while the Xa matrix showed a prominent TAD boundary at the same coordinate as the whole-population TAD boundary (Fig. 1E). This observation is consistent with previous allele-specific Hi-C studies showing that TADs are largely attenuated on Xi^8,11,12^.”*

4. The manuscript is well written.

*Reply: We thank the reviewer for the very supportive comments of our writing!*

5. At the start of the results, can you specify how many probes were used?

*Reply: We thank the reviewer for pointing this out. We now add the following details at the start of the results (Page 5): “This selected 840-kb region spans a TAD boundary, based on previously published IMR-90 HiC datasets. We partitioned this region of interest into consecutive 30-kb segments, labeled each segment with 400 primary oligonucleotide probes, and imaged each segment following our previously established chromatin tracing protocol.”*

6. Can you explain what conditional and unconditional probabilities mean? I did not understand this part. *Reply: We thank the reviewer for pointing this out. We now add an additional “Conditional and unconditional probability analyses” part in the “Methods” session to explain the conditional and unconditional probabilities (Page 28): “Conditional and unconditional probability analyses To perform conditional and unconditional probability analyses, we first ordered all triplets of chromatin segments within the traced genomic region. Chromatin segments within a distance threshold of 200-nm are defined as in contact with each other. For each triplet of segment A, B and C (ordered from 5’ to 3’ of the traced region), we define the following three probabilities. The unconditional probability between B and C is defined as the percentage of traces with B-C contact among all traces with detected B and C. The conditional probability of BC contact given AB contact is defined as the percentage of traces with B-C contact among traces with A-B contact. In this case, we first isolated all traces with A-B contact, and calculated the probability of B-C contact among these traces. The conditional probability of B-C contact given no A-B contact is defined as the percentage of traces with B-C contact among traces without A-B contact. In this case, we first isolated all traces without A-B contact, and calculated the probability of B-C contact among these traces.”*

7. Drug treatments - can you specify the length of treatments? The IF validation in Figure S1 are not convincing. First, why is there no H3K27me3 enrichment? Which cell line is this? Not female otherwise we would expect an H3K27me3 Xi-like foci? Can you quantify the signal? Most importantly, does chromatin get hyperacetylated at the region of interest? Same for H3K27me3 and transcription upon EZh2 inhibition and transcription inhibition, respectively. Without these controls it is hard to make strong conclusions about the role these marks may play at the region examined by chromatin tracing. Also α-amanitin is a transcriptional inhibitor, but it was not validated and also no conclusion was made on the role of transcription in single-cell domains. You could expand there.

*Reply: We thank the reviewer for the constructive suggestions. In terms of the lengths of drug treatments, we treated the cells for 8, 24, 48, 12 hours respectively with α-amanitin, TSA+NaBu, GSK126 and DMOG. Regarding the expected H3K27me3 enrichment, the IF images in the previous Figure S1 were indeed collected from the female cell line IMR-90. We did observe H3K27me3 enrichment on Xi but the image shown previously was not clear on this regard because we were showing one z optical slice of the cell that may or may not contain the Xi. In the revised manuscript we have re-collected H3K27me3 IF images and updated Figure S6 (previously Figure S1) to show the Xi enrichment of the epigenetic mark. We also quantified the IF signals as the reviewer requested. To test if the epigenetic perturbation occurred at the targeted genomic region of interest, we further performed combined co-IF and DNA FISH of the target region, and quantified H3K27me3 IF signals after GSK126 treatment and H3K27ac IF signals after TSA+NaBu treatment at the genomic region of interest. The results showed that the H3K27me3 levels significantly decreased after GSK126 treatment and H3K27ac levels significantly increased after TSA+NaBu treatment (Response Figure 7, new Figure S7). To test if the transcription strength was reduced after the transcription inhibition, we performed intron RNA FISH after 8 hours of αamanitin treatment and found that intron RNA FISH signals of X chromosome genes TMSB4X and RPL36A were significantly reduced (Response Figure 1, new Figure S6). As the reviewer suggested, we expanded our conclusion and now discuss epigenetic components and transcription in parallel throughout the manuscript.*

***Response Figure 7. Combined DNA FISH and immunofluorescence (IF) staining of different epigenetic perturbations.*** *Treatments with TSA + NaBu (A-B) and GSK126 (C-D) are tested. (A and C) Raw images of DNA FISH and IF staining signals. White arrows indicate DNA FISH foci. White circles indicate the IF signals at the positions of the DNA FISH foci. (B and D) Quantification of IF signal intensity at fitted DNA FISH foci positions. N = 153 and 58 for control and TSA + NaBu treated cells in B. N =104 and 128 for control and GSK126 treated cells in D. The p values were calculated using two-sided Student’s t tests.*

8. Radii of gyration. Can you explain this better? I did not understand it.

*Reply: We thank the reviewer for pointing this out. We now add the following descriptions and formula to explain the radius of gyration calculation in the “Methods” session (Page 29). “Radius of gyration calculation To calculate the radius of gyration for each chromatin trace, we measured the sum squared Euclidean distance between each traced locus to the centroid of the chromatin trace and normalized it to the number of traced loci. The radius of gyration is defined as the square-root of the normalized value, as in the formula below. In the formula, xi, yi, and zi represent the x, y, z coordinates of one target locus; xc,* *yc and zc represent the x, y, z coordinates of the trace centroid (mean x, y, z coordinates); N represents the number of traced loci. Chromatin traces with no less than 25 detected loci out of 28 targeted loci were used for radius of gyration calculation for the fine-scale chromatin tracing. Large-scale chromatin tracing data of 40 TADs across the entire human X chromosome were downloaded from a previous work^13^. Chromatin traces with no less than 35 detected loci were used for radius of gyration calculation for the large-scale chromatin tracing.*

$$Radius of gyration=\sqrt{\frac{\sum_{i=1}^{N} {(xi-xc)}^{2}+{(yi-yc)}^{2}+{(zi-zc)}^{2}}{N}}$$

*We also added the following explanations in the “Results” session (Page 11). “The radius of gyration of a trace reflects the radius of the spatial volume spanned by the trace folding conformation.”*

Additional comments:

9. Xist should be changed to its human nomenclature: XIST.

*Reply: We thank the reviewer for the correction. We now replace “Xist” to “XIST” in the manuscript.*

10. Page 4, line 26. Specify what cells are these "human female IMR-90 cell line" here (lung fibroblasts, mentioned below).

*Reply: We thank the reviewer for pointing this out. We newly added the following specifications in the manuscript (Page 5): “To investigate the chromatin folding of active and inactive X chromosomes (Xa and Xi) at the TAD-length scale in single cells, we used chromatin tracing^13,14^ to trace the chromatin folding organization of an 840-kb region of the X chromosome in a human female IMR-90 lung fibroblast cell line.”*

11. Figure 1C and 1D. Reference the study from which IMR-90 Hi-C data was reanalyzed in the panels. For example, write between brackets the published reference next to "Hi-C number of reads".

*Reply: We thank the reviewer for pointing this out. We newly added the references in the updated Figure 1C and 1D.*

12. Figure 2C and 2D. Switch 2C to 2D and 2D to 2C. Or, switch explanation in text.

*Reply: We thank the reviewer for pointing this out. We have switched the explanation in the text and pasted the updated explanation below (Page 7): “Quantification of boundary probabilities along the traced genomic region showed that in Xi, the single-cell domain boundaries had significantly more uniform distribution across the traced region (p = 2.09x10^-4^, F-test) (Fig. 2C-D), leading to the lack of strong ensemble boundary in Xi (Fig. 1E), as the single-cell domain boundaries across this genomic region are “averaged out” during the ensemble calculation. In Xa, however, single-cell domain boundaries preferentially resided at the ensemble TAD boundary (Fig. 2D), leading to a prominent ensemble boundary after population averaging (Fig. 1E).”*

13. Page 6, line 21-22. Explain Xi first and Xa later (following the order of the figures).

*Reply: We thank the reviewer for pointing this out. We now change the explanation as below (Page 9): “Xi showed attenuated TAD boundary whereas Xa showed strong ensemble TAD boundary (Fig. S5A-B). Individual Xi and Xa traces showed similarly frequent and strong single-cell domains (Fig. S5C-H). These observations show that the single-cell domains on Xi and Xa are not unique to the IMR-90 cells.”*

14. Figure 3. This figure should be merged with figure 2 (or shown as supplemental figure) since it does not make a new point but rather extends figure 2 findings to another human cell line.

*Reply: We thank the reviewer for the suggestion. We agree with the reviewer and have changed Figure 3 into Supplementary Figure 5.*

15. Figure S1. This figure should show the immunofluorescence images in the order that appear on the text TSA + NaBu, DMOG and GSK126.

*Reply: We thank the reviewer for pointing this out. We have changed the order of the drug treatment conditions in the updated Supplementary Figure 6 (previously Figure S1).*

16. Figure 4A-D. Add the treatment applied to each panel in the figure. In addition, add examples for both X chromosome per condition.

*Reply: We thank the reviewer for pointing this out. In the revised manuscript, we have added the treatment conditions to the figure panels and added examples for both Xa and Xi for each condition in the updated Figure 3 (previous Figure 4).*

17. Figure 4 E-F. DMOG seems to increase boundary frequency and strength, could you check if this increase is non-significant or significant? Also, run statistical tests for all conditions.

*Reply: We thank the reviewer for pointing this out. We performed two-sample t-test to compare boundary frequency and boundary strength between DMOG treatment and control conditions. The DMOG treatment indeed led to a minor but statistically significant increase in boundary frequency (p =1.55x10^-5^ and 8.30x10^-8^ for Xi and Xa, respectively) and boundary strength (p =6.80x10^-3^ and 1.14x10^-9^ for Xi and Xa, respectively). We also performed statistical tests for all conditions and found that none of the other drug treatment conditions significantly affected boundary frequencies and boundary strengths (p > 0.01 for other drug treatment conditions). We have added the following description to the manuscript regarding the minor but significant increase of boundary frequency and strength with the DMOG treatment (Page 10): “The DMOG treatment led to a minimal increase of both the boundary frequency and boundary strength, whereas none of the other treatments significantly affected the appearance, boundary frequencies, or strengths of the single-cell domains in Xi or Xa (p>0.01 with Bonferroni correction) (Fig. 3B-F, S6). The small increase of boundary frequency and strength under the DMOG treatment could be due to secondary effects of the drug.”*

18. Figure 4 E-F. GSK126 treated cells do not have allelic resolution information. Why? Can you please add allelic resolution to this condition?

*Reply: We thank the reviewer for pointing this out. We previously failed to get allelic resolution for GSK126 treated cells because the XIST signal significantly deceased after the treatment, making the Xi/Xa assignment difficult. We newly collected GSK126-treated datasets using mH2A1 staining for Xi/Xa assignment and updated Figure 3F-3G (previous Figure 4E-4F) with allelic information.*

19. Page 6. Perturbation experiment, similar to point above. Add information about which cell line and how long these inhibitors were applied.

*Reply: We thank the reviewer for pointing this out. We now add the information in the main text (Page 9): “First we treated IMR-90 cells for 24 hours with a combination of histone deacetylase (HDAC) inhibitors trichostatin A (TSA) and sodium butyrate (NaBu) that lead to hyperacetylation of histones (Fig.S6A), given that histone acetylation prevents nucleosome compaction. Surprisingly, the single-cell domains persisted in both Xi and Xa under the drug treatment with similar boundary frequency and strength as in the untreated control (Fig. 3). We further treated IMR-90 cells for 12 hours with dimethyloxalylglycine (DMOG) - an HIF-prolylhydroxylase inhibitor (Fig. S6B), which causes a global increase of histone methylation, for 48 hours with GSK126, an enhancer of zeste homolog 2 (EZH2) methyltransferase inhibitor (Fig. S6C), for 8 hours with α-amanitin, an RNA polymerase inhibitor (Fig. S6D), and for 24 hours with 5-aza-2’- deoxycytidine (ZdCyd), a DNA methylation (cytosine-C5) inhibitor (Fig. S6E).”*

*20. Page 6, line 34-35. Clearly indicate that both (TSA and NaBu) inhibitors are added together.*

*Reply: We thank the reviewer for pointing this out. We now add the information in the main text in the updated paragraph above.*

21. Page 6. Perturbation experiment. Can you include a DNA methylation perturbation experiment to ask if DNA methylation can affect single-cell domains?

*Reply: We thank the reviewer for the suggestion. To perturb DNA methylation, we treated cells with 5-aza-2′-deoxycytidine (ZdCyd), a DNA methylation (cytosine-C5) inhibitor for 24 hours. We then performed IF staining to validate the decrease in cytosine-C5 DNA methylation (Figure S6). We further analyzed chromatin conformations of the targeted region and found that the appearance, boundary probability and boundary strength of Xa and Xi single-cell domains were not affected by the inhibition of DNA methylation (Figure 3E-3G). These results were newly added to the manuscript.*

22. Figure 5. Indicate in panel A and B from where these radii of gyration come from (single TADs versus TAD-to-chromosome). Fig. 5B. Add between brackets on the y-axis the study from which this data was re-analyzed (reference 30).

*Reply: We thank the reviewer for the suggestion. We newly added the corresponding information in the updated Figure 5.*

23. Discussion: Can you hypothesized which molecular mechanism could affect single-cell domains in the discussion?

*Reply: We thank the reviewer for the suggestion. We now add the following discussions of hypothetical molecular mechanisms that could affect single-cell domains, including a previous hypothesis and new hypotheses that we propose (Page 11-12):*

*“Loop extrusion and epigenetic landscape have been shown to be major, parallel contributing factors to the spatial organization of TADs^15,16^. As the disruption of loop extrusion did not abolish single-cell domains4 , recent studies proposed that epigenetic interactions mediate the formation of single-cell domains and demonstrated such possibility with computational modeling^17,18^. To recapitulate the highly variant single-cell domains, the modeling involves several constant epigenetic profiles (termed “binding sites” in the model) across a genomic region; different epigenetic reader proteins (termed “binders” in the model) dynamically bind to the corresponding epigenetic binding sites and can mediate the interactions between pairs of genomic loci with the same kind of epigenetic binding sites; the thermodynamics of the chromatin polymer leads to contacts between genomic loci, some of which are captured by the epigenetic interactions mediated by the binders; due to the thermodynamic nature of the system, the distribution of the chromatin contact instances and the epigenetic interaction instances differ from one copy of the polymer to another, which underlies the different single-cell domain structures in the model^17,18^….” “Due to the limitation of this study, we cannot rule out the possibility that some untested epigenetic components, including various histone and DNA modifications and non-histone DNA binding proteins, underlie the formation of single-cell domains. Neither can we rule out the possibility that the tested epigenetic components are compensated by other epigenetic components. In addition, Xa and Xi-specific allelic profiles of the tested epigenetic components in the IMR-90 cell line are lacking. Whether our observations can be generalized to the entire X chromosome or the whole genome is also unknown. Thus this work does not disprove the epigenetic model. However, our data suggest that alternative explanations for the formation of single-cell domains may deserve equal consideration. For example, an unknown motor protein other than cohesin may drive the formation of single-cell domains through loop extrusion. Alternatively, some nuclear components may form scaffolds or aggregates that push away chromatin into adjacent pockets of free nuclear space that correspond to single-cell domains. Further studies may reveal the mechanism underlying the single-cell domains, as well as their dynamics and functional significance in single cells.”*

24. Discussion: Discuss your results in light of these recent pre-prints: 1) Bauer/Payer 2020 bioRxiv (TAD-like compartmentalization persists on the Xi) and 2) Takei/Cai 2021 bioRxiv (single-cell chromosome domain structures on Xa and Xi chromosomes) both studies performed in mouse.

*Reply: We thank the reviewer for the suggestion. We now add the following discussions of the two papers (Pages 11 and 12): “In this work, we showed that ensemble TADs on the inactive X chromosome were attenuated (but not completely lost) whereas TADs on the active X chromosome were prominent, consistent with previous findings in mouse^8–10^ .”*

*“Strikingly, the active and inactive X chromosomes, despite having distinct epigenetic states^5,19,20^, both can contain single-cell domains of similar features. This observation did not appear to depend on a particular human cell type or on the open or closed states of the chromatin region. A concurrent preprint also reported the observation of single-cell domains on both Xa and Xi chromosomes in mouse brain tissue^21^, supporting the prevalence of these structures.”*

Reviewer #3:

The authors used chromatin tracing to analyze the genome organization in human female cells. They compared the active and inactive X chromosomes. They found TAD-like single cell domains in both Xa and Xi cells despite the drastic difference between the Xi and Xa chromosomes at the bulk level: TADs are observed in Xa but not in Xi chromosome. These single cell domains are highly variable across cells. The authors repeated these experiments in two different cell types (IMR90 and hTERT-RPE-1 cells) to confirm that this observation is not specific to one particular cell type. Furthermore, they applied several inhibitors to perturb histone modifications and transcription. They found that the single cell domains are resistant to such perturbations. They also showed that the Xi is more compact than Xa at the large but not fine scale. This study reports an interesting and exciting observation. This manuscript can be further improved by addressing the following issues. 1. As the single cell domains exist for both Xa and Xi, more discussion is needed to explain why Xa shows TADs but Xi not at the bulk level. There is one sentence in the paper to state that the Xi single domain boundaries are more uniformly distributed while the Xa boundaries occur at the TAD boundaries. This statement requires a quantitative measurement to support it.

*Reply: We thank the reviewer for the suggestion. To better explain the reason, we now add the following texts in the paper (Page 7):*

*“Quantification of boundary probabilities along the traced genomic region showed that in Xi, the single-cell domain boundaries had significantly more uniformed distribution across the traced region (p = 2.09x10^-4^, F-test) (Fig. 2C-D), leading to the lack of strong ensemble boundary in Xi (Fig. 1E) as the single-cell domain boundaries across this genomic region are “averaged out” during the ensemble calculation. In Xa, however, single-cell domain boundaries preferentially resided at the ensemble TAD boundary (Fig. 2D), leading to a prominent ensemble boundary after population averaging (Fig.1E).” As shown above, to quantitatively support the argument that the Xi single-cell domain boundaries are more uniformly distributed, we newly performed two-sample F-test to compare the variance of boundary probabilities along the traced genomic region between Xi and Xa for all three chromatin tracing target regions in IMR-90 and also the original target region in RPE-1 cells. The results showed that in all cases, the variances of boundary probabilities significantly differ between Xi and Xa (p<0.05). In IMR-90, p=2.09x10^-4^ for the original region, p=0.026 for the new closed region, p=0.0098 for the new open region. In RPE-1, p=1.87x10^-4^ for the original region. These quantifications are now added to the corresponding texts and figures.*

2. Related to the above point, do cells fall into different clusters with similar domain patterns? The variation analysis of the single cell domains can be done at the cluster level if there are distinct clusters. Showing bulk TAD boundaries would also be helpful to illustrate the variation of the single cell domains.

*Reply: We thank the reviewer for the insightful suggestion. We now add the following contents in the manuscript (Page 8) to address the reviewer’s comment on the clusters and attach the new Figure S1 (Response Figure 8) below. We have also added yellow lines to mark ensemble TAD boundaries in the mean spatial distance matrix plots (Fig. 1B, 1E, 4C, S1B, S3A, S4A, S5A-B) and in the single-cell domain matrix plots (Fig. 2A-B, 3A-E, S3B-C, S4B-C, S5C-D) to illustrate the variations of the single-cell domains as the reviewer suggested.*

*“To further analyze the structural differences between Xi and Xa, we performed LouvainJaccard clustering of individual chromatin traces, using each chromatin trace as a highdimensional data entry and the pairwise distances between the 30-kb target regions as variables. We found that chromatin traces formed clusters with different conformations (Fig. S1A-B). Each conformational cluster had a mixture of Xi and Xa traces, indicating that Xi and Xa both had similarly diverse conformations (Fig. S1C-D). Clusters had different ratios of Xi and Xa traces (Fig. S1D), consistent with the different ensemble chromatin conformations of Xi and Xa.”*

***Response Figure 8. Conformational clustering of chromatin traces.*** *(****A****) Louvain-Jaccard clustering result of chromatin trace conformations displayed with t-distributed stochastic neighbor embedding (t-SNE). Each dot represents a chromatin trace. Different pseudo-colors represent different chromatin trace clusters. (****B****) Mean spatial distance matrices of the traced genomic region for six major clusters identified in A. The yellow lines represent the ensemble TAD boundary. (****C****) The same t-SNE plot of chromatin traces as in A but colored by Xi (blue) and Xa (red) identities. (****D****) Ratio of chromatin trace copy numbers of Xi and Xa in the six major clusters in B.* *N = 960 for A, C and D. Genomic target region is the same as in Fig. 1.*

3. Are CTCF and cohesin enriched at the single cell domain boundaries?

*Reply: We thank the reviewer for the suggestion. We downloaded the CTCF- and cohesion core subunit RAD21-binding narrow peak calls from ENCODE consortium1 and analyzed their distributions along the traced genomic region. The analyses showed that in Xi, single-cell domain boundaries do not seem to strongly correlate with the CTCF and RAD21 peaks; in Xa, two CTCF and RAD21 peaks do appear to coincide with frequent single-cell domain boundaries, one of which is also the ensemble TAD boundary (Response Figure 9, part of new Figure 2).*

*Response Figure 9. Single-cell domain boundary frequencies in Xi and Xa and the CTCF and RAD21 binding peaks across the traced genomic region.*

4. The interpretation of the epigenetic inhibitor results requires some caution. These inhibitors affect the global histone methylation or acetylation. But to draw a concrete conclusion that epigenetic modification or transcription does not affect single cell domains requires mapping histone modifications in single cells for Xi and Xa after inhibitor treatment. It would also be interesting to cluster the single cell domains after perturbation and compare them with the untreated cells.

*Reply: We thank the reviewer for the suggestion. Indeed as the reviewer pointed out, although our new time-course IF measurements (Fig. S6) and co-DNA FISH and IF measurements (Fig. S7) validated the global effects of the inhibitors and supported a local effect at the targeted genomic region, our data is limited by the lack of allelic epigenetic profiles of Xi and Xa in the IMR-90 cell line. To clarify this limitation and caution our readers, we now add the following discussion of this and other limitations of our study in the manuscript (Page 12):*

*“Due to the limitation of this study, we cannot rule out the possibility that some untested epigenetic components, including various histone and DNA modifications and non-histone DNA binding proteins, underlie the formation of single-cell domains. Neither can we rule out the possibility that the tested epigenetic components are compensated by other epigenetic components. In addition, Xa and Xi-specific allelic profiles of the tested epigenetic components in the IMR-90 cell line are lacking. Whether our observations can be generalized to the entire X chromosome or to the whole genome is also unknown. Thus this work does not disprove the epigenetic model.”*

*As the reviewer suggested, we further performed cluster analysis of the single-cell domains after perturbations and compared them with the untreated cells. We now add the following contents in the manuscript (Page 10) and show the clustering and comparison results in the new Fig. 4 (Response Figure 10):*

*“To further resolve the influences of different epigenetic perturbations on chromatin conformations, we combined chromatin traces from all epigenetic perturbation conditions and control, and performed Louvain-Jaccard clustering of the traces as in Fig. S1. A total of six conformational clusters were revealed (Fig. 4A). The clusters do not correspond to different epigenetic perturbations, as chromatin traces from different perturbations and control were highly dispersed among the clusters (Fig. 4B). Mean spatial distance matrices of the clusters showed distinct chromatin conformations (Fig. 4C). Different epigenetic perturbations and control were roughly equally represented in each cluster (Fig. 4D). Taken together, these analyses indicated that the perturbations of several major epigenetic components and transcription did not systematically alter the diverse chromatin conformations of the traced X chromosome region.”*

***Response Figure 10.*** ***Clusters of chromatin conformations under perturbations of epigenetic components and transcription machinery.*** *(A) Louvain-Jaccard clustering of chromatin traces displayed with t-distributed stochastic neighbor embedding (t-SNE). Each dot represents a chromatin trace. Different pseudo-colors represent different identified clusters. (B)* *The same t-SNE plot of chromatin traces as in A but colored by different perturbation conditions. (C) Mean spatial distance matrices of the traced genomic region for the six chromatin trace clusters identified in A. The yellow lines represent the ensemble TAD boundary. (D) Ratio of chromatin trace copy number in one cluster over that in all clusters for each perturbation (and control).* *N = 2856 for A, B and D. Genomic target region is the same as in Fig. 1.*

5. The imaging data should be available for download.

*Reply: We thank the reviewer for the suggestion. Over the past several years we have been attempting to find a viable solution to address the challenge of sharing the raw imaging data from our chromatin tracing works. Unfortunately despite our multiple attempts listed below, we have not found a viable solution. The challenge is, for example, the raw images for the submitted analyses in this work is over 21 TB (not GB) in size. Our school does not offer us a server of this size, and does not allow us to set up a publicly accessible server by ourselves to share this data due to the medical school patient data network security and protection regulations and laws (even though our data contains no patient information). We cannot find any external data sharing service that can host such size of data (and offer the necessary internet bandwidth for downloading such data) without incurring unbearable cost to us. Recently we discussed with the NIH 4DN consortium data sharing center. Even though the center is highly dedicated to data sharing within the consortium and to the public, they do not offer hosting such large raw image data, but only host analyzed spreadsheet-type data. In a sense, our imagebased spatial genomics data are similar to sequencing-based omics data, where people usually cannot routinely share raw spot images from sequencers due to the prohibitive large size (and often these raw images from the sequencers are not even kept in sequencing facilities). People usually only upload base calling results from sequencing for sharing. It seems the whole imagebased omics community is still in the process of developing a solution for this important task as the reviewer pointed out. To partial address this issue, we have made our analyzed data publicly accessible through this following link and added this information to the revised manuscript: https://campuspress.yale.edu/wanglab/GenomeBiology2021. For groups interested in our raw image data, we can offer to physically mail hard drives containing our data upon request.*

Minor: 1. reference genome in Methods and Table S1 is hg18. Is it hg38?

*Reply: We thank the reviewer for the question. The reference genome in Methods and Table S1 is hg18, not hg38.*

*References*

*1. The ENCODE Project Consortium. An integrated encyclopedia of DNA elements in the human genome. Nature 489, 57–74 (2012).*

*2. Jin, F. et al. A high-resolution map of the three-dimensional chromatin interactome in human cells. Nature 503, 290–294 (2013).*

*3. Davis, C. A. et al. The Encyclopedia of DNA elements (ENCODE): data portal update. Nucleic Acids Res. 46, D794–D801 (2018).*

*4. Bintu, B. et al. Super-resolution chromatin tracing reveals domains and cooperative interactions in single cells. Science 362, eaau1783 (2018).*

*5. Galupa, R. & Heard, E. X-Chromosome Inactivation: A Crossroads Between Chromosome Architecture and Gene Regulation. Annu. Rev. Genet. 52, 535–566 (2018).*

*6. Dixon, J. R. et al. Topological domains in mammalian genomes identified by analysis of chromatin interactions. Nature 485, 376–380 (2012).*

*7. Rao, S. S. P. S. P. et al. A 3D Map of the Human Genome at Kilobase Resolution Reveals Principles of Chromatin Looping. Cell 159, 1665–1680 (2014).*

*8. Bauer, M. et al. Chromosome compartments on the inactive X guide TAD formation independently of transcription during X-reactivation. Nat. Commun. 12, 3499 (2021).*

*9. Wang, C.-Y., Jégu, T., Chu, H.-P., Oh, H. J. & Lee, J. T. SMCHD1 Merges Chromosome Compartments and Assists Formation of Super-Structures on the Inactive X. Cell 174, 406- 421.e25 (2018).*

*10. Giorgetti, L. et al. Structural organization of the inactive X chromosome in the mouse. Nature 535, 575–579 (2016).*

*11. Minajigi, A. et al. Chromosomes. A comprehensive Xist interactome reveals cohesin repulsion and an RNA-directed chromosome conformation. Science 349, aab2276- (2015).*

*12. Deng, X. et al. Bipartite structure of the inactive mouse X chromosome. Genome Biol. 16, 152 (2015).*

*13. Wang, S. et al. Spatial organization of chromatin domains and compartments in single chromosomes. Science 353, 598–602 (2016).*

*14. Hu, M. & Wang, S. Chromatin Tracing: Imaging 3D Genome and Nucleome. Trends in Cell Biology vol. 31 5–8 (2021).*

*15. Rao, S. S. P. et al. Cohesin Loss Eliminates All Loop Domains. Cell 171, 305–320 (2017).*

*16. Schwarzer, W. et al. Two independent modes of chromatin organization revealed by cohesin removal. Nature 551, 51 (2017).*

*17. Conte, M. et al. Polymer physics indicates chromatin folding variability across single-cells results from state degeneracy in phase separation. Nat. Commun. 11, 1–13 (2020).*

*18. Esposito, A. et al. Polymer physics and machine learning reveal a combinatorial code linking chromatin 3D architecture to 1D epigenetics 2. bioRxiv 2021.03.01.433416 (2021) doi:10.1101/2021.03.01.433416.*

*19. Lessing, D., Anguera, M. C. & Lee, J. T. X chromosome inactivation and epigenetic responses to cellular reprogramming. Annu. Rev. Genomics Hum. Genet. 14, 85–110 (2013).*

*20. Pasque, V. & Plath, K. X chromosome reactivation in reprogramming and in development. Curr. Opin. Cell Biol. 37, 75–83 (2015). 21. Takei, Y. et al. Integrated spatial genomics in tissues reveals invariant and cell type dependent nuclear architecture. http://biorxiv.org/lookup/doi/10.1101/2021.04.26.441547 (2021) doi:10.1101/2021.04.26.441547.*

**Second round of review**

**Reviewer 1**

The authors addressed most of my questions. I only have some minor suggestion remains as the following.

(1) It would be much informative to have Figu3. F and G separated with xa and xi.

(2) It is interesting that xi and xa showed the similar dynamic in the boundaries, however, this conclusion may specific to the region tested. In this revision, author added two more regions in Figure S3, S4, which is nice. But I think it is worth to have a plot shows the same statistic as in Figure S1.D included to these two newly added regions.

(3) Same to the question (2), I also like to see the statistics for those perturbations (Figure4)

**Reviewer 2**

This manuscript uses chromatin tracing to determine the chromatin structure of the active and inactive X chromosomes at the level of single cells. They found that the inactive X retained some boundary structure at the level of single cells, but unlike the active X these boundaries were distributed unbiasedly across the region. Perturbing transcription and various epigenetic marks did not have a significant effect on these single cell domains. The authors added analysis of two additional regions, which increases the applicability of the results to the wider X chromosome state compared with the previous submission. Additional perturbation experiments and statistical analyses were also performed. I found that the reviewers answered the reviewers comments in a satisfactory manner.

Additional comments:

1. Could the authors comment more on why the original region was picked? Is there any significance to this region? For each of the three regions analyzed, could a map of the genes in the region be included , possibly on figure S2?

2. Figure 2g: Is the thick black line the unconditioned probability? In the figure legend it appears more grey. This should be fixed to make it more clear.

3. Figure 3 legend: The authors have added the p-values for their comparisons to the figure legend. It would be easier to compare these if they were formatted in a table or included in the figure. The epigenetic target of each inhibitor could also be included in the figure or table for easy reference.

**Reviewer 3**

The authors have addressed all the comments.

**Authors Response**

**Point-by-point responses to the reviewers’ comments:**

Reviewer #1: The authors addressed most of my questions. I only have some minor suggestion remains as the following. (1) It would be much informative to have Figure3. F and G separated with xa and xi.

*Reply: We thank the reviewer for the suggestion. We have separated Xa and Xi in the updated Figure 3F and Figure 3G.*

(2) It is interesting that xi and xa showed the similar dynamic in the boundaries, however, this conclusion may specific to the region tested. In this revision, author added two more regions in Figure S3, S4, which is nice. But I think it is worth to have a plot shows the same statistic as in Figure S1.D included to these two newly added regions.

*Reply: we thank the reviewer for the suggestion. To study whether Xi and Xa show similar conformation dynamics in the two newly added regions, we performed LouvainJaccard clustering of chromatin traces of the two regions. For both newly added regions, each conformational cluster had a mixture of Xi and Xa traces, indicating that Xi and Xa had similarly diverse conformations. We now add the newly analyzed results in Figure S5 and Figure S6.*

(3) Same to the question (2), I also like to see the statistics for those perturbations (Figure4).

*Reply: We thank the reviewer for the suggestion. To study whether Xi and Xa show similar conformation dynamics under different drug perturbation conditions, we plotted the ratios of Xi and Xa in each drug perturbation condition and found that Xi and Xa traces were similarly mixed in each chromatin trace cluster under all drug perturbation conditions. We now add the newly analyzed results in Figure S10.*

Reviewer #2:

This manuscript uses chromatin tracing to determine the chromatin structure of the active and inactive X chromosomes at the level of single cells. They found that the inactive X retained some boundary structure at the level of single cells, but unlike the active X these boundaries were distributed unbiasedly across the region. Perturbing transcription and various epigenetic marks did not have a significant effect on these single cell domains. The authors added analysis of two additional regions, which increases the applicability of the results to the wider X chromosome state compared with the previous submission. Additional perturbation experiments and statistical analyses were also performed. I found that the authors answered the reviewers comments in a satisfactory manner.

Additional comments:

1. Could the authors comment more on why the original region was picked? Is there any significance to this region? For each of the three regions analyzed, could a map of the genes in the region be included, possibly on figure S2?

*Reply: We thank the reviewer for the suggestion. The original region was picked based on previously published Hi-C data, and was selected solely because the region covered a distinct TAD boundary on the Hi-C heatmap. We have newly included maps of genes in the three regions on Figure S2 as the reviewer* *instructed.*

2. Figure 2g: Is the thick black line the unconditioned probability? In the figure legend it appears more grey. This should be fixed to make it more clear.

*Reply: We thank the reviewer for pointing this out. We have updated Figure 2G as instructed.*

*3. Figure 3 legend: The authors have added the p-values for their comparisons to the figure legend. It would be easier to compare these if they were formatted in a table or included in the figure. The epigenetic target of each inhibitor could also be included in the figure or table for easy reference.*

*Reply: we thank the reviewer for the suggestion. We have newly added a table summarizing all adjusted p values in Figure 3H. We also added the epigenetic target of each inhibitor in Figure 3F.*
